# Contribution of Network Connectivity in Determining the Relationship between Gene Expression and Metabolite Concentration Changes

**DOI:** 10.1371/journal.pcbi.1003572

**Published:** 2014-04-24

**Authors:** Aleksej Zelezniak, Steven Sheridan, Kiran Raosaheb Patil

**Affiliations:** 1 European Molecular Biology Laboratory, Heidelberg, Germany; 2 Technical University of Denmark, Kgs. Lyngby, Denmark; Ecole Polytechnique Fédérale de Lausanne, Switzerland

## Abstract

One of the primary mechanisms through which a cell exerts control over its metabolic state is by modulating expression levels of its enzyme-coding genes. However, the changes at the level of enzyme expression allow only indirect control over metabolite levels, for two main reasons. First, at the level of individual reactions, metabolite levels are non-linearly dependent on enzyme abundances as per the reaction kinetics mechanisms. Secondly, specific metabolite pools are tightly interlinked with the rest of the metabolic network through their production and consumption reactions. While the role of reaction kinetics in metabolite concentration control is well studied at the level of individual reactions, the contribution of network connectivity has remained relatively unclear. Here we report a modeling framework that integrates both reaction kinetics and network connectivity constraints for describing the interplay between metabolite concentrations and mRNA levels. We used this framework to investigate correlations between the gene expression and the metabolite concentration changes in *Saccharomyces cerevisiae* during its metabolic cycle, as well as in response to three fundamentally different biological perturbations, namely gene knockout, nutrient shock and nutrient change. While the kinetic constraints applied at the level of individual reactions were found to be poor descriptors of the mRNA-metabolite relationship, their use in the context of the network enabled us to correlate changes in the expression of enzyme-coding genes to the alterations in metabolite levels. Our results highlight the key contribution of metabolic network connectivity in mediating cellular control over metabolite levels, and have implications towards bridging the gap between genotype and metabolic phenotype.

## Author Summary

Regulation of metabolic activity in response to environmental and genetic perturbations is fundamental to the growth and maintenance of all cells. A primary regulatory process used by cells to control the activity of their metabolic network is the alteration in the expression of enzyme-coding genes. How these alterations regulate metabolite concentrations is an important question in the quest towards unraveling the genotype-phenotype relationship. The link between the expression levels of enzymes and metabolite concentrations is governed by the kinetics of individual reactions, which in turn are interlinked with each other due to the complex connectivity structure of metabolic networks. Although the enzyme-metabolite relationship is relatively well studied at the level of individual reactions, our understanding of the regulation of metabolite levels in complex networks has remained incomplete. In this study, we show that the constraints imposed by the network connectivity are key determinants of the relationship between gene expression and metabolite concentration changes. Our results provide mechanistic insight into the function of complex metabolic networks and have implications for health and biotechnological applications.

## Introduction

Cellular metabolic networks provide basic biochemical building blocks and a thermodynamically favorable environment for growth and maintenance. Due to this crucial role of metabolism, cells have evolved various mechanisms to regulate metabolic reactions in response to genetic and environmental changes. Metabolic reactions can be regulated either by modulating the availability of the corresponding enzymes, e.g. through altered transcription and/or translation, or, by modulating the enzyme activities through post-translational modifications or through binding of small molecules. Our knowledge of the landscape of transcriptional, translational and post-translational regulation of metabolism is expanding with the increasing availability of datasets that provide genome-wide views of the abundance and interactions between mRNAs, proteins and metabolites [1]–[6]. Although the relative contribution of each of these regulatory layers is still unclear and is likely to be context dependent, it has long been clear that the adjustments in the cellular metabolic phenotype (i.e., rates of reactions, or fluxes, and metabolite levels) often involve changes at the level of gene expression [7]–[9]. For example, previous studies have shown that the gene expression changes in metabolic networks are centered on metabolites that are crucial for adjusting the network state in response to specific perturbations [10], [11]. Despite successful outcomes of these and other studies suggesting a strong link between transcriptional regulation and changes in metabolite levels [7], [10], [12], [13], the relationship between the two has remained elusive.

**Figure 1:**
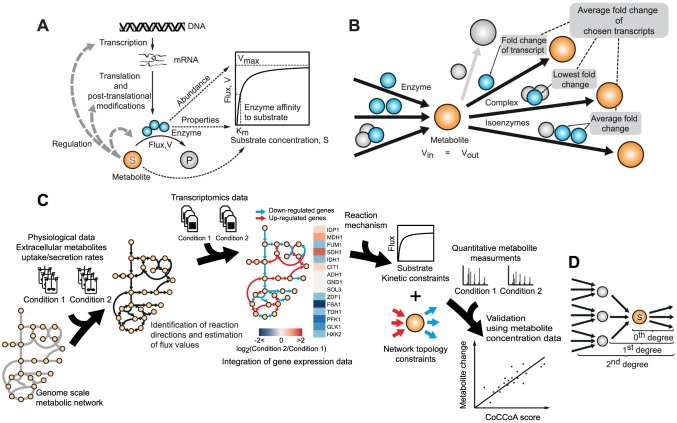
From gene expression to metabolite levels. A) Metabolite levels are only indirectly affected by changes in gene expression levels, through changes in the corresponding enzyme abundances. Metabolites, in turn, can provide feedback to the regulatory network controlling enzyme abundance/activity. B) Transcript-metabolite relationships are usually many-to-one. We discarded transcripts with insignificant changes (P≤0.05) (grey circles). For the remaining transcripts we combined the corresponding fold changes to derive gene-expression scores for reactions and thereby for consumption or production of metabolites (see Main text). C) Schematic workflow used for the proposed concentration change coupling analysis (CoCCoA). In the first step, physiological measurements from growth experiments are used to constrain the genome-scale metabolic model (Methods). Subsequent flux simulations (Methods) help in identifying the directionality and range of fluxes under the conditions being compared. Next, by using the comparative transcriptome data, fold changes at the individual gene-expression level are mapped on to the reactions in the network (panel B). CoCCoA integrates the mapped gene-expression data with the network topology by using a model formulation derived from the MM reaction kinetics mechanism and mass balance constraints (main text). The main output from the algorithm is a measure of transcriptional control over metabolite levels, or the CoCCoA scores, which are tested for correlation with the experimentally measured metabolite concentration changes. D) Schematic representation of three different CoCCoA models with varying degrees of network connectivity constraints.

The task of developing models for describing the relationship between gene expression and metabolite concentrations is challenging due to the multiple layers of regulation involved in between (Figure 1A). Several of the regulatory mechanisms involved, such as translational control or allosteric regulation, are currently poorly understood at the scale of the whole network. Measurement of protein abundances or enzyme activities is also currently difficult to perform at the network scale and in complex systems such as human tissues. Thus, in the absence of data for intermediate molecular players, a detailed investigation of the link between gene expression and metabolite levels has both a fundamental and a practical appeal. In particular, it is of interest to estimate the degree to which the changes at the level of gene expression affect changes in metabolite concentration and to uncover the underlying mechanisms determining their relationship. In this study, we explore the hitherto poorly understood role of network connectivity constraints in controlling metabolite concentrations in a eukaryotic model organism, *Saccharomyces cerevisiae*. We postulate that two primary mechanisms will largely determine the association between the changes in mRNA and metabolite levels: reaction kinetics (which are non-linear by nature [14]–[16]) and the mass balance constraints imposed by the network, i.e. the balance between production and consumption of metabolites. Although we here focus on mRNA levels due to genome-wide coverage of the available transcriptomics datasets, the proposed model can also be readily applied to enzyme abundance or activity data.

The role of reaction kinetics in controlling metabolite concentration has been previously examined mostly from the perspective of the isolated reaction-metabolite pairs. With such a reaction-centric approach, a previous study on yeast metabolism was able to partially explain changes in the intracellular metabolite levels when using protein abundance as a measure of enzyme availability [3]. However, no correlation was observed in the same study when using gene expression data instead of protein abundances. One possible reason for the lack of strong correlation between gene expression and metabolite levels when looking at the isolated enzyme-metabolite pairs is that the large connectivity inherent to metabolic networks is not taken into account. A large fraction of intra-cellular metabolites participate in multiple reactions. For example, over 25% of the yeast metabolites participate in more than three reactions [17]. Consequently, abundance of an enzyme catalyzing a particular reaction cannot completely determine the concentrations of the participating metabolites or the rate of the reaction. Indeed, correlations between mRNA and fluxes, and even between enzyme activities and fluxes, have been often found to be poor [18]–[21]. Approaches accounting for the network connectivity of metabolites have been successful in linking gene expression to metabolites in an empirical or qualitative manner [10], [12], [22]–[24], but have achieved only a limited success on the quantitative front. Advantages of both reaction-centered kinetics approaches and network topology-based approaches can be combined in network kinetic models that include detailed kinetics of all involved reactions [25]–[28]. However, application of kinetic models to large metabolic networks is difficult due to their reliance on a large number of parameters. Such parameters are either currently unavailable, or their estimation requires comprehensive measurements of intra-cellular states of interest (e.g. metabolite concentrations, enzyme abundances, and fluxes) in the vicinity of the perturbation to be modeled.

In this study, we propose a steady-state model of the transcriptional control of metabolite concentrations. Our model integrates reaction kinetics and metabolic network connectivity constraints without requiring the knowledge of kinetic parameters. In essence, the model uses mass balance constraints to bridge the individual reaction kinetic constraints with those of the other reactions in the network. The resulting equations provide a log-linear relationship between the fold-change in the concentration of a given metabolite to the fold-changes in the expression of its neighboring genes, as well as topologically more distant genes.

## Results

By analogy to flux coupling analysis [29], which describes how steady-state fluxes are linked with each other, we termed our approach Concentration Change Coupling Analysis (CoCCoA). Starting with a classical reaction kinetics model, which treats each reaction as an isolated system consisting of a single enzyme and its substrate, we developed a network kinetics approach by accounting for the interactions between different reactions through their shared metabolites. As there is currently a lack of information on *in vivo* enzyme kinetics mechanisms at the network-scale, we used the single-substrate Michaelis-Menten (MM) kinetics for all reactions. In essence, MM kinetics describes the flux or reaction rate *V* as a function of three parameters: i) concentration of the substrate, *S*; ii) maximum capacity of the enzyme pool, *V*_max_; and iii) a parameter reflecting the enzyme's kinetic properties, *K_M_* (Figure 1A). The central idea of CoCCoA is to use mass balance constraints on the flux term *V* to link single-reaction kinetics to the other reactions in the network. We considered MM kinetics in the fold-change space, which allowed us to eliminate the need to know the *K_M_* values. For each metabolite, CoCCoA provides an overall transcriptional change score (CoCCoA score) according to the CoCCoA equations, which are developed in the subsequent sub-sections. To assess the proportion of variance in metabolite changes that can be attributed to transcriptional regulation, we compared the calculated CoCCoA scores with the experimentally measured metabolite concentration changes. The overall workflow used is depicted in Figure 1B.

The first step in our analysis is to calculate a representative transcriptional fold-change for each reaction. As the yeast metabolic network consists of several reactions that are each governed by multiple proteins, we classified all reactions into three types: i) reactions catalyzed by a single enzyme, ii) reactions catalyzed by two or more isoenzymes, and iii) reactions catalyzed by enzyme complexes. We then applied the following rules to calculate the representative fold-changes for all reactions: in the case of isoenzymes, we averaged the fold changes of the related transcripts, while in the case of complexes, we picked the transcript with the lowest fold change (Figure 1C). We used only significantly changed transcripts (P-value≤0.05) in the presented analysis. Relaxation of this filtering criterion did not change the overall results (Figure S1).

### Experimental datasets and the metabolic network

We used four published experimental datasets for evaluating the proposed CoCCoA models. These case studies included three pairwise comparisons – one genetic [3] and two environmental perturbations [23], [30] – and a time-course dataset obtained during the yeast metabolic cycle [9], [31]. In all pair-wise comparison studies, both gene expression and metabolite concentration data were obtained from the same experiment. In the case of the metabolic cycle data, although the sampling for transcriptome and metabolome was performed in two separate experiments, the experimental setups were identical and the sampling was performed at comparable time-points spanning all phases of the metabolic cycle. While the metabolic cycle is fundamentally a non-steady-state phenomenon, the observed transcript oscillation period of about 300 minute means that a reasonable degree of pseudo-steady-state can be assumed for applying our model. In the case of the three pairwise comparison studies, the correlations between CoCCoA scores and metabolite concentrations provided a perturbation-centered perspective wherein the responses of different metabolites were analyzed jointly. The metabolic cycle case study allowed us to additionally evaluate the gene expression-metabolite relationship from a metabolite-centered perspective, wherein the response of each metabolite was assessed individually for its conformity to the proposed model.

A genome-scale metabolic reconstruction of *S. cerevisiae* [17] was used to obtain the metabolite-reaction-gene connectivity information and to estimate the reaction directionalities (Methods). For each case study, we used the experimental measurements of exchange fluxes (uptake and secretion rates of metabolites) to constrain and simulate a flux balance model. Accordingly, we removed all blocked reactions and reactions for which the flux directions could not be unambiguously assigned. We also excluded the reactions for which the predicted flux directions did not agree between the two conditions being compared.

Depending on the extent to which the network connectivity information is included in the calculations, we term the CoCCoA models as 0^th^ degree, 1^st^ degree, 2^nd^ degree, and so on (Figure 1D). 0^th^ degree CoCCoA relies on the enzyme kinetics alone and thus considers only the consumption reaction(s) of any given metabolite. 1^st^ degree CoCCoA additionally considers the production of the metabolite by using mass balance constraints. 2^nd^ degree CoCCoA further expands the degree of network connectivity accounted for in the model by including the producing reactions of the precursors of the metabolite in question. Alternatively, the 2^nd^ and higher degree models can also be expanded on both the consumption and production sides of the metabolite as described in the following sub-sections (also see Text S1).

### 0^th^ degree concentration change coupling analysis

We consider metabolite concentration changes relative to a reference condition that can be arbitrarily chosen from the conditions pertaining to the experiment under investigation. Assuming that the enzyme properties (represented by *K_M_*) remain unchanged in the experiment, by using MM kinetics one obtains (Text S1):
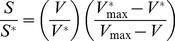
(1)


Where ^*^ denotes the reference condition. The relative nature of this formulation allows circumventing the problem of the lack of availability of *in vivo K_M_* values. Furthermore, by assuming that 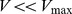 *&* 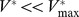, and that the ratio 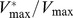 can be approximated by the gene expression ratio, equation (1) simplifies to a log-linear relationship (equation (2), Text S1). Both of these assumptions are critically examined in the next sub-section. The model represented by equation (2) is hereby termed *0^th^ degree coupling*, meaning that the metabolite *S* is not considered to be directly coupled to any other metabolite and is connected only to the enzyme that uses it as a substrate (Figure 1D).
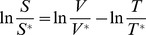
(2)


### Evaluation of non-saturation and mRNA-protein correlation assumptions

The first assumption used in deriving equation (2) implies that the enzyme is not saturated. The opposite situation, i.e. an enzyme approaching saturation, is not amenable for establishing the metabolite-gene expression relationship (or metabolite-enzyme abundance relationship in general), as the reaction velocity will then be only a weak function of the substrate concentration. Recent studies have shown that *in vivo* concentrations for several metabolites, especially from central carbon metabolism, are close to the corresponding *K_M_* values [32]. At these concentrations, reaction rates *V* are close to half of the *V_max_*. Although the assumption of *V≪V_max_* is not strictly applicable in this flux regime, numerical simulations showed modest errors (around 20%) due to this approximation (Figure S2). Moreover, if the saturation level does not change drastically between the two conditions being compared, the error remains close to zero (Figure S2). Given the advantage that this approximation brings, namely elimination of the need for knowing the *in vivo* kinetic parameters, the cost of the approximation error appears to be acceptable.

**Figure 2:**
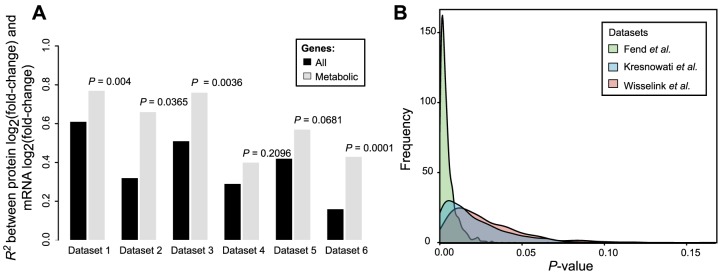
Changes in mRNA and protein abundances are strongly correlated for metabolic genes. A) Shown are the coefficients of determination for mRNA fold-change – protein fold-change correlations; black: with all proteins measured in each of the datasets, gray: only with metabolic proteins. *P-*values denoting the significance of the improved correlations for metabolic genes were estimated based on random sampling from the set of measurements including all proteins. B) Distributions of *P*-values obtained for 1^st^ degree CoCCoA analysis while accounting for the variability in the correlations between the mRNA and protein fold-changes.

The second major assumption is that the fold-change in mRNA level can be used as a proxy for the fold-change in enzyme abundance and ultimately for the fold-change in *V_max_*. Critical examination of this assumption is of particular importance as the role of translational efficiency and post-translational modifications in regulating metabolic enzymes is becoming increasingly evident [18], [33]–[36]. We examined our assumption by analyzing published experimental data for *S. cerevisiae* where genome-wide mRNA and protein fold changes were simultaneously measured. In support of the assumption, the correlations between the mRNA and the protein fold changes corresponding to the metabolic genes were found to be both significant and strong (Dataset 1–3 [37], [38], R^2^ = 0.77, *P* = 0.04; R^2^ = 0.66, *P* = 0.0365, R^2^ = 0.76, *P* = 0.0036; dataset 4 [39], R^2^ = 0.4, *P* = 0.296; dataset 5 [40], R^2^ = 0.57, *P* = 0.0681; dataset 6 [41], R^2^ = 0.43, *P* = 0.0001) (Figure 2A). We note that these correlations involving only metabolic genes are stronger than the correlations calculated by including the non-metabolic genes (Figure 2A, Figure S3). As mRNA and protein levels have recently been demonstrated to be in good agreement in mammalian systems as well [42], we expect that the assumption of proportionality between gene expression and protein abundance fold changes will be valid in a broad range of organisms.

### Evaluation of 0^th^ degree concentration change coupling analysis

**Figure 3:**
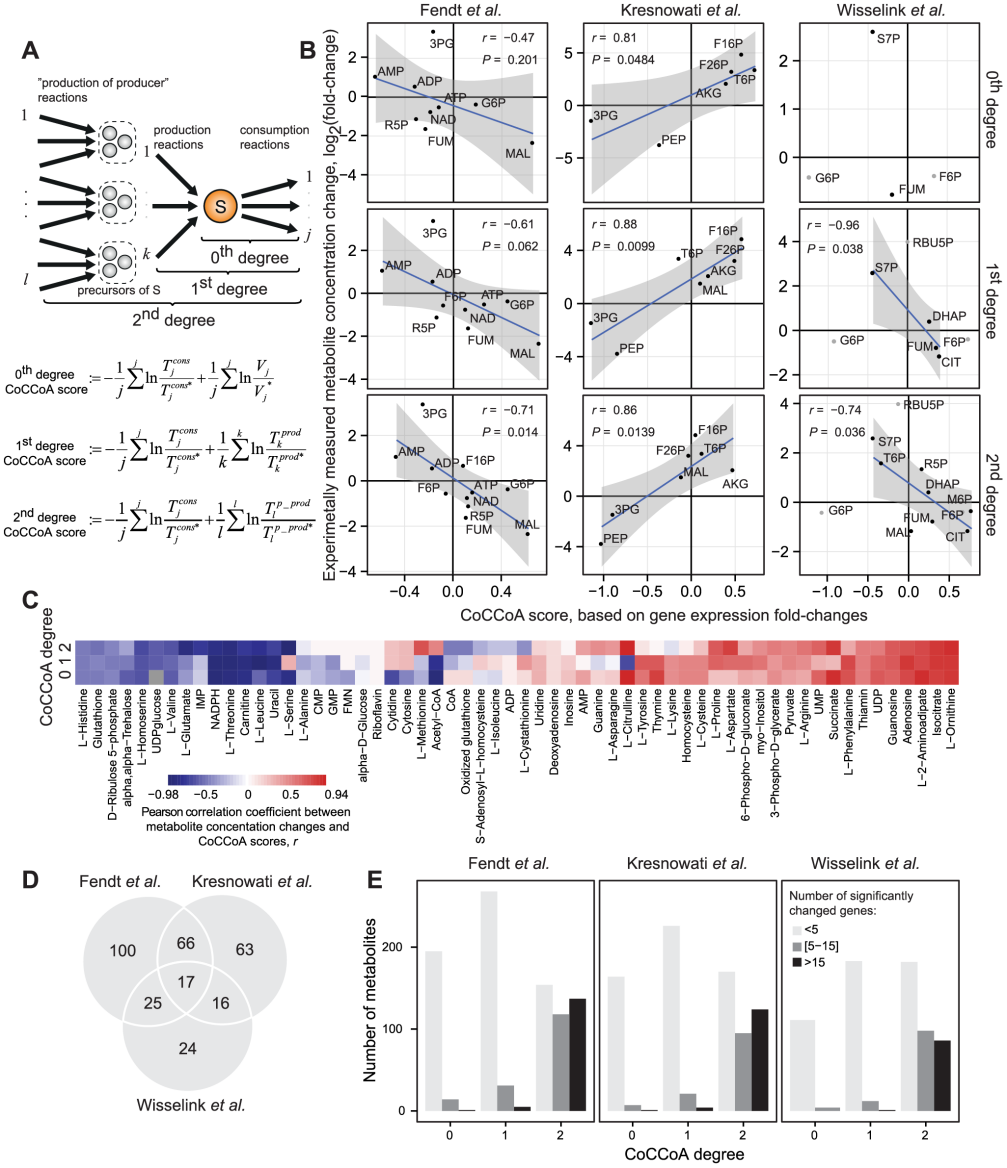
Metabolite concentration changes are correlated with CoCCoA scores. A) Equations illustrating the calculations of CoCCoA scores of different degrees (Main text, Figure S5). B) Correlations between experimentally measured metabolite concentration changes and CoCCoA scores for the three pairwise comparison datasets [3], [23], [30]. Note that the number of data points that can be tested varies for each coupling degree as the number of genes included in the analysis changes with the degree of the CoCCoA equation (see main text). Metabolites marked in gray could not be included in the analysis as the directions of the fluxes linked to them were altered between the growths on two different carbon sources [30]. C) Pearson correlation coefficients comparing experimentally measured metabolite concentration changes and CoCCoA scores for the metabolic cycle study [9]. D) Overlap between the significantly expressed genes in the three pairwise comparison case studies. Only genes that were used for the calculation of CoCCoA scores are included. E) Number of genes used in the CoCCoA score calculations increase with the increasing model degree. 3PG = 3-Phospho-D-glycerate; G6P = Glucose 6-phosphate; F26P = Fructose 2,6-bisphosphate; F16P = Fructose 1,6-bisphosphate; FUM = Fumarate; CIT = Citrate; MAL = Malate; AKG = alpha-Ketoglutarate; T6P = Trehalose 6-phosphate; PEP = Phosphoenolpyruvate; S7P = Sedoheptulose 7-phosphate; RBU5P = Ribulose 5-phosphate; R5P = Ribose 5-phosphate; DHAP = dihydroxyacetone phosphate; M6P = Mannose 6-phosphate.

Under the condition of flux homeostasis, i.e. no flux change between the two conditions being compared, the metabolite concentration ratio in equation (2) becomes dependent only on the transcript change. The resulting 0^th^ degree CoCCoA model is equivalent to the analysis of the transcript/protein-metabolite relationship reported by Sauer and co-workers [3]. According to this model, we observed a significant correlation between transcriptional and metabolite changes in the glucose pulse case study (*r* = 0.81, *P* = 0.048). In the other two pairwise comparison case studies, the 0^th^ degree model failed to correlate with the experimentally observed metabolite changes (Figure 3B). In case of the metabolic cycle dataset, around 31% of the measured metabolites showed significant correlations (FDR 10%) (Figure 3C, Figure S1). In all four case studies investigated here, flux homeostasis cannot be assumed as the growth rate as well as the substrate uptake and product secretion rates were affected by the corresponding perturbations. Our attempts to obtain reliable flux estimates by using flux balance analysis were not fruitful since only a limited number of physiological measurements were available to constrain the model, resulting in a high degree of uncertainty in the flux estimates. Thus, in the absence of reliable intra-cellular flux estimates, the 0^th^ degree model was found to be insufficient for relating gene expression changes to metabolite levels.

### 1^st^ degree concentration change coupling analysis

At steady state, the sum of fluxes producing a particular metabolite must be equal to the sum of fluxes through the reactions that use it as a substrate. For a metabolite with a single production reaction and a single consumption reaction, the steady state assumption combined with equation (2) leads to equation (3) (Supplementary Text S1).
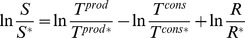
(3)


*T^prod^* and *T^cons^* denote expression levels of the genes corresponding to the enzymes producing and consuming *S*, respectively. *R* refers to the concentration of the metabolite that is the precursor of *S*. The relation described by equation (3) implies a coupling between the concentration changes in *R* and *S*, and is here defined as 1^st^ degree coupling. In comparison to the 0^th^ degree coupling, the flux term ln(V/V*) is eliminated in the 1^st^ degree coupling equation and is replaced by two new terms, ln(T^prod^/T^prod*^) and ln(R/R^*^). Equation (3) brings a new network perspective to enzyme kinetics, whereby gene expression and metabolite concentration changes in the adjacent reactions are linked through the mass balance constraint. Each metabolite pool is thus linked to the reactions consuming it as well as on the reactions producing it (Figure 3A). When multiple reactions are consuming (or producing) the same metabolite *S*, the consumption (or production) term can be approximated by the geometric mean of the transcript ratios of all the consumption (or production) reactions (Supplementary Text S1).

To evaluate the 1^st^ degree model, we compared the experimentally measured metabolite concentration ratios with the 1^st^ degree CoCCoA scores based on the transcript fold changes – the first two terms on the right-hand side of equation (3). We note that, although the strict application of our model requires the use of the ln(R/R^*^) term, these measurements are often not available. Moreover, a model that is completely independent of the metabolite concentration data will likely be of more practical value. Omitting the ln(R/R*) term equates to assuming that the preceding metabolite's concentration does not change between the two conditions; an alternative to omitting this term is explored below, in the 2^nd^ degree CoCCoA model, in which the ln(R/R*) term is estimated by use of the 1^st^ degree CoCCoA model. The effect of omitting the ln(R/R^*^) term on CoCCoA scores is further discussed in the later sub-section “*Post-transcriptional regulation*”.

Comparisons of the 1^st^ degree CoCCoA scores to metabolite concentrations yielded significant correlations in the two environmental perturbation case studies (*r* = 0.88, *P* = 0.0099 and *r* = −0.96, *P* = 0.038), and a reasonably good correlation (*r* = −0.61, *P* = 0.06) for the genetic perturbation case study (Figure 3B, Figure S1). For the metabolic cycle case study, around 25% of the measured metabolites showed significant correlations (FDR 10%) (Figure 3C, Figure S1). Although our model suggests positive correlation between CoCCoA scores and metabolite changes, we observed negative correlations in the cases of two of the pairwise comparisons and for some of the metabolites in the metabolic cycle case study. The possible reasons underlying this discrepancy are discussed in the subsequent sub-section “*Negative correlations in CoCCoA*”. We maintain that the negative slopes do not invalidate the significance of the observed correlations, but rather hint at the existence of unaccounted parameters/mechanisms leading to the reversal of slope in some cases.

The number of transcripts that can be used for the calculation of the CoCCoA scores typically increases as more distant reaction nodes in the network are included with the increasing CoCCoA degree. Consequently, the number of metabolites that could be assigned CoCCoA scores varied between the coupling degrees. For example, in the C-source change study [30] (Figure 3B), only 4 metabolites have significant transcript changes corresponding to their consuming reactions and hence only these could be compared against the experimental data for the 0^th^ degree analysis. In contrast, 2^nd^ degree CoCCoA scores could be calculated for 7 metabolites.

### Network propagation of concentration control

In a similar manner as going from the 0^th^ to the 1^st^ degree coupling, the CoCCoA equations can be further extended to include more distant nodes in the metabolic network. By replacing the concentration ratio in the right-hand side of Equation 3 (i.e. R/R*) with the 1^st^ degree CoCCoA relationship for the corresponding precursor metabolite (in this case, *R*), we obtained the 2^nd^ degree coupling relationship. This 2^nd^ degree model accounts for the gene expression changes corresponding to the precursor's production reactions (Figure 3A) (Text S1). In all case studies, the 2^nd^ degree correlations remained as strong as for the 1^st^ degree. This result is notable since the 2^nd^ degree coupling score includes expression data from the genes that are further away from the metabolites in question. With further extension of the CoCCoA model in a similar manner, we observed significant correlations up to the 6^th^ degree coupling (*P*≤0.05, Fendt *et al.* case study, Figure S4).

**Figure 4:**
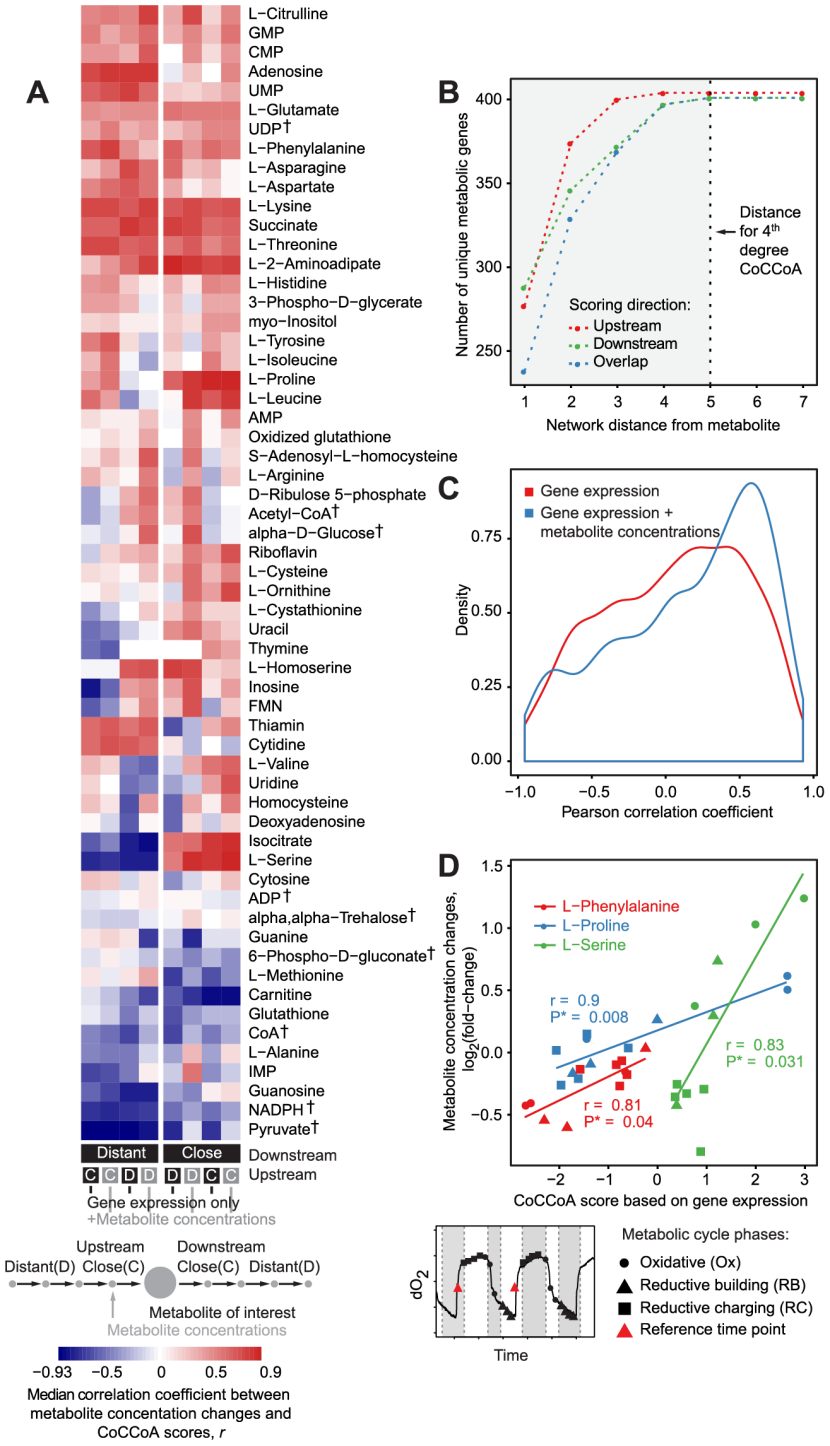
CoCCoA models link the changes in gene expression and metabolite concentrations during yeast metabolic cycle. A) Pearson correlation coefficients comparing the experimentally observed metabolite concentration changes and CoCCoA model scores. For a given metabolite, 16 different CoCCoA scores were computed, accounting for a network distance of up to 4, both upstream and downstream of the metabolite in question. Scores were divided into two sets: close (distance ≤2) and distant (distance >2). Shown values are the median correlation coefficients in each set. B) CoCCoA models account for a large fraction of genes in the metabolic network at relatively short distances. C) Correlations between the experimentally observed metabolite concentration changes and CoCCoA model scores improve with the inclusion of the concentrations of the precursors and/or products of the metabolite in question. D) Example correlations between the CoCCoA model scores and the metabolite concentration changes. Different shapes of data points mark different stages of the yeast metabolic cycle. † - Metabolites with previous evidence for post-translational regulation of at least one of their neighboring enzymes [34].

To gain further insight into the metabolite concentration control at different network distances, we examined this problem from a metabolite-centric perspective by taking advantage of the broad metabolite coverage of the metabolic cycle case study. First, we extended the higher degree CoCCoA formulation so as to include information from all intermediate reaction steps up to the desired degree (Text S1). For example, the calculation of the 3^rd^ degree CoCCoA score includes fold changes from the genes associated with the reactions involving all metabolites that are three steps upstream or downstream from the metabolite in question. The inclusion of genes within a desired network distance can either be restricted to the consumption or the production side of the metabolite, or both included simultaneously. This formulation also allowed us to include, if available, measured concentrations of the neighboring metabolites within the desired distance of a given metabolite, and thereby to assess the effect of changes in neighboring metabolites over its concentration. The algorithm used for calculating CoCCoA scores using this formulation is described in Text S1. In brief, this algorithm first enumerates all paths starting from the metabolite of interest to identify genes that are within a given network distance. Next, it uses graph topology-based heuristics to weight and incorporate the expression fold-changes corresponding to these genes into the CoCCoA equations by using mass balance considerations. Following this, we evaluated the ability of these CoCCoA scores to explain the concentration changes observed during the metabolic cycle. Overall, positive correlations are apparent for most of the metabolites (∼66%, Figure 4A,C). For the long distance scores, a slightly lower number of metabolites showed positive correlations. The contrast between the close and distant neighbors, however, should be interpreted in light of the highly connected nature of the metabolic network. The numbers of genes that are included in the calculation of CoCCoA scores already reach a plateau at the 4^th^ degree (Figure 4B), and thus, even relatively modest distances can mean inclusion of a very large fraction of the network. Consequently, the CoCCoA scores for a given metabolite will become ‘diluted’ due to the noise stemming from the inclusion of gene expression changes pertaining to the reactions that are only indirectly affecting the metabolite of interest. These results from the metabolic cycle case study, together with the results from the pairwise comparison studies, suggest that the close neighbors in the metabolic network exert the majority of the control over metabolite concentrations.

The correlations between the CoCCoA scores and the metabolite concentration changes were further strengthened when the experimentally determined fold-changes in the concentration of the upstream and/or downstream metabolites were also used in the calculation (Figure 4C). This improvement further supports the CoCCoA theory, as the inclusion of concentration changes for the upstream/downstream metabolites stems from the joint mass balance and kinetic considerations, e.g. as illustrated in equation (3).

### Limitations of CoCCoA formulation

CoCCoA is not applicable in the case of perturbations that are likely to drastically affect the kinetic properties (*K_M_* values) of several enzymes in the network, or if the metabolite concentrations are considerably above the corresponding *K_M_* values (saturated enzymes). Furthermore, the CoCCoA model needs to exclude reactions for which the flux directionality is ambiguous, and it assumes that the flux directions do not change for the rest of the reactions. Post-translational regulatory mechanisms, which can affect the kinetic parameters, are also not included in the current CoCCoA formulation, as sufficient data are not available to enable their modeling. The latter is perhaps the most restrictive limitation of our model. Post-translational regulation is known to play a crucial role in the yeast central metabolism, wherein several enzymes are controlled by allosteric binding of small molecules [35], [43], [44] and/or through post-translational modifications such as phosphorylation [33]. Together, these various assumptions and limitations can lead to poor or no correlations. In the case of the pairwise comparisons, poor correlations can also result from the pooling of metabolites with positive and negative correlation with CoCCoA scores into a single plot.

According to the CoCCoA model, all examined correlations would be expected to be positive. Among the pairwise comparison case studies, positive correlations were observed only for the glucose pulse study (Figure 3B). In the case of the metabolic cycle study, a significant majority of the metabolites (∼66%, *P* = 2.9×10^−8^, exact binomial test) showed positive correlations (Figure 4A,C). Flux regulation due to allosteric binding by small molecules and post-translational modifications are likely to be the major factors underlying this discrepancy between the expected positive slopes and the observed negative slopes for the remaining 34% metabolites. The possible causes and implications of negative correlation are discussed in the subsequent sub-section “*Negative correlations in CoCCoA*”.

### Robustness of CoCCoA towards differences in gene expression and protein abundance fold changes

We found that the enzyme-coding genes in yeast exhibit significantly stronger correlations between mRNA and protein fold-changes than do the non-metabolic genes (Figure 2A, Figure S3). The slopes of these correlations were, however, different across different datasets examined. To evaluate the robustness of CoCCoA towards this variation, we re-performed 1^st^ degree CoCCoA analysis multiple times (1000 simulations), adjusting the transcript ratios in each simulation by a randomly sampled correction factor to account for the expected difference between the mRNA and protein fold changes. The sampling space for the correction factors was estimated based on the variance in the slopes of linear regression lines between mRNA and protein abundance fold changes across different datasets (Figure S3, Text S1). We then examined the number of simulations in which the correlation between the 1^st^ degree CoCCoA scores and the metabolite fold changes remained significant. For all three pairwise comparison case studies, the correlations remained significant (P≤0.05) in a large fraction of these simulations (99%, 86% and 91%) (Figure 2B).

## Discussion

### Network constraints over metabolite concentration changes

The correlation between metabolites concentrations and transcript fold changes becomes evident only when including network connectivity constraints (1^st^ and higher degree CoCCoA). Thus, inclusion of gene expression changes associated with the both upstream and downstream reactions was critical for explaining metabolite concentration changes. For all three pairwise comparison case studies, CoCCoA models explained more than 60% of the variation in metabolite changes based on gene expression. For the metabolic cycle case study, CoCCoA could explain variation in about 33% of the measured metabolites, with correlation coefficients as strong as 0.90.

The use of a genome-scale metabolic model was crucial in CoCCoA analysis in order to capture the large connectivity inherent to metabolic networks. Even for a sparsely connected metabolite such as D-Ribose 5-phosphate, for which the 0^th^ degree score accounted for only 4 transcripts, the 2^nd^ degree CoCCoA score accounted for transcriptional information from 47 genes in the Fendt *et al.* study [3] (Figure 3B). With the increasing degree of CoCCoA equations, larger numbers of genes become part of the CoCCoA score (Figure 3E, Figure 4B). We also observed that, in general, the inclusion of new genes when moving from the 1^st^ to the 2^nd^ degree CoCCoA maintains the significance of the correlation (Figure 3B, Figure 3C). This observation implies strong co-regulation of genes that are linked through common substrates/products. Indeed, co-expression of metabolic genes at short network distances has been observed in earlier studies [45]. The CoCCoA theory suggests that homeostasis of metabolite concentrations is one of the objectives of such topology-oriented co-regulation in metabolic networks.

### Post-transcriptional regulation

There are several layers of regulation that the CoCCoA model does not take into account: translational and post-translational regulation, and the kinetic effects of neighboring metabolites' concentrations. With regard to translational regulation, we observed strong correlations between transcript and protein fold changes (Figure 2A, Figure S3), and the CoCCoA results were found to be relatively robust in light of the known variability (Figure 2B). However, it remains to be seen whether these correlations extend through post-translational regulation to enzyme activity. Increasing availability of the genome-wide protein phosphorylation/acetylation data may aid in addressing this question. The CoCCoA framework can be used with enzyme abundance or enzyme activity fold changes in place of transcript fold changes. Using this information could lead to more accuracy in CoCCoA scores, and could furthermore aid in identifying the relative contribution of the different regulatory layers in controlling metabolite concentrations.

The effects of neighboring metabolites' concentrations could be examined thanks to the large coverage of metabolite measurements in the metabolic cycle dataset. The power of CoCCoA in explaining variance in metabolite concentration changes was substantially improved following the inclusion of data for the neighboring metabolites, representing ln(R/R^*^) terms in the CoCCoA equations (2.54 fold increase in the median correlation coefficient; *P* = 6.6×10^−8^, Wilcox test; Figure 4C). In accordance with the CoCCoA theory, this improvement indicates that a metabolite's neighbors in the metabolic network play an important role in determining its level.

### CoCCoA is applicable to perturbations of different nature

Formulation of CoCCoA in a relative manner, i.e. in the fold-change space, allowed us to circumvent the problem of the unavailability of *in vivo* kinetic parameters. A major advantage following this relative formulation is that the CoCCoA models do not need any parameter fitting. Indeed, the applicability of CoCCoA was found to be quite broad in terms of the perturbation or experimental design underlying the data. The four datasets considered in this study represent three different biological perturbations, namely gene knockout [3], nutrient pulse [23], change in carbon source [30], as well as a fundamental rhythmic phenomenon associated with the cell cycle [9], [31]. These case studies also span two distinct cultivation types, batch [3] and chemostat [9], [23], [30], [31]. We also verified the differences in the nature of these perturbations at the level of gene expression changes: the three pairwise comparison studies were found to have only a small overlap in terms of the significantly responding genes (Figure 3D). Additionally, the CoCCoA model was found to be applicable over a broad range of concentration changes displayed by different metabolites during the yeast metabolic cycle (Figure 4D).

### Negative correlations in CoCCoA

Intriguingly, several of the observed negative correlations were found to be not only significant but also quite strong, with *R^2^* values as high as 0.86 (Figure 4). These negative correlations are indicative of the mechanisms that are unaccounted for in the CoCCoA model and/or highlight cases in which the assumptions of the model do not apply. We observed that the number of positive correlations in the metabolic cycle case study increased considerably when using the concentration change data from the neighboring metabolites (the ln(R/R^*^) term in the right-hand side of the 1^st^ degree CoCCoA equation) (Figure 4C). This observation suggests that the kinetic effect due to changes in the neighboring metabolites is an important factor contributing to negative correlations. The results from the analysis of the metabolic cycle data also hint that the negative slopes might be characteristic to certain metabolites, for example, those for which the producing/consuming enzymes are regulated predominantly and/or prevalently at the post-translational level. Indeed, we found that the metabolites with poor or negative correlations in the metabolic cycle case study are enriched in the metabolites with previous evidence for post-translational regulation of at least one of their neighboring enzymes (metabolites marked with † in Figure 4A, data for post-translationally regulated enzymes from [34], *P* = 0.0006). In these cases, the post-translational regulation may be counteracting the transcriptional change. Post-translational regulation of an enzyme can change its *K_M_* value and can thereby directly affect CoCCoA scores. Consider, for example, 1^st^ degree CoCCoA score. When changes in *K_M_* values are included in the 1^st^ degree CoCCoA formula, the score becomes: 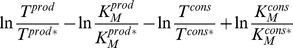. If the post-translational modifications counteract the transcriptional changes, the *K_M_* ratios in this new score will partially cancel out or even override the transcript ratios. When assuming constant *K_M_* values, the discrepancy between the transcript and *K_M_* ratios in some cases might be sufficiently large to result in CoCCoA scores with opposite sign. On the other hand, the inconsistency between the directions of transcriptional and post-translational regulation (or post-transcriptional regulation in general) implies non-optimal regulation and is unlikely to be a general mechanism used by the cell. However, non-optimal regulation is a possibility for certain enzymes, with two plausible biological explanations: i) the highly non-linear scenario of regulation (resulting from the concerted action of reaction kinetics, incl. allosteric regulation, and mass balance and thermodynamic constraints) can mean that the cell needs to make some locally non-optimal choices in order to achieve a global optimality in regulating the overall metabolism (for example, to take advantage of the distinct time-scales at which post-translational and transcriptional regulations act); ii) the observed behavior is both locally and globally non-optimal in case of certain perturbations. The second scenario would imply that the perturbations in question are unknown or new to the cells in the evolutionary sense.

In addition to the unaccounted post-transcriptional regulation, the simplifying assumptions of constant flux directions and flux split ratios may also be contributing to the observed negative correlations. A wrongly considered flux direction for a reaction would mean that the corresponding fold change in the expression level would be treated in the opposite direction. Similarly, moderate changes in the flux split ratio can also cause a sign reversal in the CoCCoA score if one of the fluxes is significantly lower than the other(s). The interaction between the various missing/simplifying factors in our model can further amplify the difference between the CoCCoA scores and actual concentration changes. How these interactions lead to the reversal of correlation while retaining statistical significance is yet unclear. Further investigation into the mechanisms underlying these intriguing negative correlations would require network-wide *in vivo* measurements of fluxes, metabolite concentrations, protein abundances and functional post-translational modifications. Nevertheless, we note that our model revealed significant correlations in several cases, including perturbations of very different nature. We also note that, in the case of metabolic cycle dataset, significantly more positive correlations were observed than negative (*P* = 2.9e-08, exact binomial test). Together, the statistical and mechanistic considerations suggest that the CoCCoA model captures considerable mechanistic essence of the complex processes governing metabolite levels.

Overall, our model-guided analysis highlighted the role of metabolic network connectivity in modulating metabolite concentration changes and revealed much stronger correlations between gene expression and metabolite levels than previously appreciated. The proposed model can be extended to include translational and post-translational regulation as the data becomes available. From this perspective, we see CoCCoA as a framework with a strong mechanistic yet parameter-free basis, rather than a general relationship. We anticipate that, due to its parameter-free nature, the CoCCoA framework will be widely applicable for modeling metabolite level changes in large metabolic networks.

## Methods

### Experimental datasets

Four different experimental studies reporting gene expression and metabolite concentration measurements for the yeast *S. cerevisiae* were used. The first study, Fendt *et al.* [3], includes a comparison between wild type yeast and a mutant strain lacking *GCR2*, a transcription factor responsible for activation of glycolytic genes [46]. In the second study, Kresnowati *et al.* [23], yeast cultures were grown in carbon-limited chemostat cultures and subjected to a step change in glucose concentration. In the third study, Wisselink *et al.* [30], an evolutionarily engineered strain was grown on either glucose or arabinose as the sole carbon source. In the fourth study, oscillating chemostat cultures were sampled covering different phases of the metabolic cycle [9]. A summary of the growth conditions and descriptions of datasets from all case studies is provided in Table S1. Metabolite data was used as available in the original studies; a significance cut-off α = 10% was chosen to control for the type 1 error. For the metabolic cycle data, we considered metabolites with the periodicity P-value≤0.05 as reported in the original study [31]. As the datasets used did not distinguish between the cytosolic and mitochondrial concentration of metabolites, we regarded all metabolite concentrations as cytosolic. Since 2-phosphoglycerate and 3-phosphoglycerate are usually indistinguishable in the MS measurements, we considered only 3-phosphoglycerate gene neighbors and excluded 2-phospoglycerate.

### Metabolic network and flux variability analysis

A genome-scale reconstruction of *Saccharomyces cerevisiae* metabolic network by Forster *et al.* [17] was used to map metabolite-reaction-gene connectivity. For each of the case studies, the functional reaction directions of reversible reactions were estimated by using flux variability analysis [47]. For this purpose, the model was constrained with the physiological data obtained from the publications reporting the used datasets (Table S2, Table S3, Table S4, and Table S5) [3]. Linear programming problems were solved using the *glpk* solver (http://www.gnu.org/software/glpk/) accessed through a C library.

### Transcription data analysis

Preprocessing of the Affymetrix CEL files was carried out with the statistical software environment R/Bioconductor (www.bioconductor.org). Probe intensities were corrected for background by using robust multi-array average method (RMA) [48] using only perfect-match probes, and normalization was performed using the quantiles algorithm. Gene expression intensity values were calculated from the perfect-match probes with median polish summarization method [49]. Significance of the differential expression was calculated by using the empirical Bayes test as implemented in the *limma* package [50].

### Statistical analysis

Pearson correlation coefficients between log_2_ metabolite fold changes and CoCCoA scores were calculated with the statistical software R (www.r-project.org) using the function *cor.test()*. Metabolite changes were used as dependent variables and CoCCoA scores as independent. *P*-values for the null hypothesis of no correlation (regression slope = 0) were estimated by using the same function. In addition, we performed a permutation test by shuffling gene labels before calculating CoCCoA scores. The originally paired data was randomly permuted without replacement 1000 times. For each permutation, a correlation coefficient was calculated and the *P*-value was estimated as a fraction of squared correlation coefficients that were larger than in the case of the original paired data. The results were similar to those estimated with the cor.test() function.

### Code used for CoCCoA score calculations

http://www.patil.embl.de/supplementary

## Supporting Information

Figure S1 Correlations between experimentally measured metabolite concentration changes and CoCCoA scores based on gene expression fold changes. A) Facet columns correspond to three different pairwise comparison datasets used in our work; rows represent different CoCCoA models. B) Heatmap of Pearson correlation coefficients assessing the applicability of CoCCoA models to the metabolic cycle study. In the present figure, the significance thresholds for the transcript and metabolite fold changes are relaxed in comparison to the data shown in Figure 3B,C in the main text.
(PNG)

Figure S2 Estimates of error in predicting metabolite concentration changes with MM kinetics when assuming V≪Vmax (see main text for the motivation behind the use of this assumption). **A**. Error (Z-axis) as a function of V/V_max_ and V_*_/V_max_^*^. The error function is shown in supplementary Text S1 (equation 11). **B**. Error estimates around the points where V/V_max_ = V_*_/V_max_^*^, representing a situation in which the enzyme saturation levels remain unchanged in the perturbed condition. **C**. 2-D projection of the plot in **A**, where the errors are represented with different colors.
(PNG)

Figure S3 Correlation between protein abundance changes and the corresponding mRNA abundance changes is stronger for metabolic proteins. A, D, G, J, M, P) Correlation including all proteins measured in different datasets. B, E, H, K, N, Q) Correlations including only metabolic proteins (as per genome-scale metabolic model by [17]). C, F, I, L, O, R) Histogram of 10,000 different correlation coefficients obtained for randomly chosen protein-transcript pairs (number of chosen pairs for each correlation being equal to the number of metabolic proteins measured in the corresponding dataset). Blue area denotes the number of random correlations that were higher than those obtained for the correlation based on the actual data. Each row of plots represents a different dataset (from top to bottom), 1, 2, 3 –[38], [51]; 4 –[41]; 5 –[40]; 6 –[41].
(PNG)

Figure S4 Coefficients of determination for the correlations between experimentally measured metabolite concentration changes and CoCCoA scores corresponding to different degrees. The significance of correlations was assessed against correlations obtained with random permutations of gene labels.
(PNG)

Figure S5 Example CoCCoA score calculations. Shown is the case of fumarate in the Fendt *et al.* case study.
(PNG)

Table S1 Summary of the growth conditions from the three pairwise comparison case studies used in our analysis.
(DOCX)

Table S2 Physiological data from the pairwise comparison case study 1.
(DOCX)

Table S3 Physiological data from the pairwise comparison case study 2.
(DOCX)

Table S4 Physiological data from the pairwise comparison case study 3.
(DOCX)

Table S5 Reaction directions used for the case studies 1 and 2.
(DOCX)

Text S1 Supporting text.
(DOCX)
