# Supplementary figures and images for: Contribution of Network Connectivity in Determining the Relationship between Gene Expression and Metabolite Concentration Changes
Source: PLoS Comput Biol. 2014 Apr 24;10(4):e1003572. doi: 10.1371/journal.pcbi.1003572 (PMC3998873; doi:10.1371/journal.pcbi.1003572)

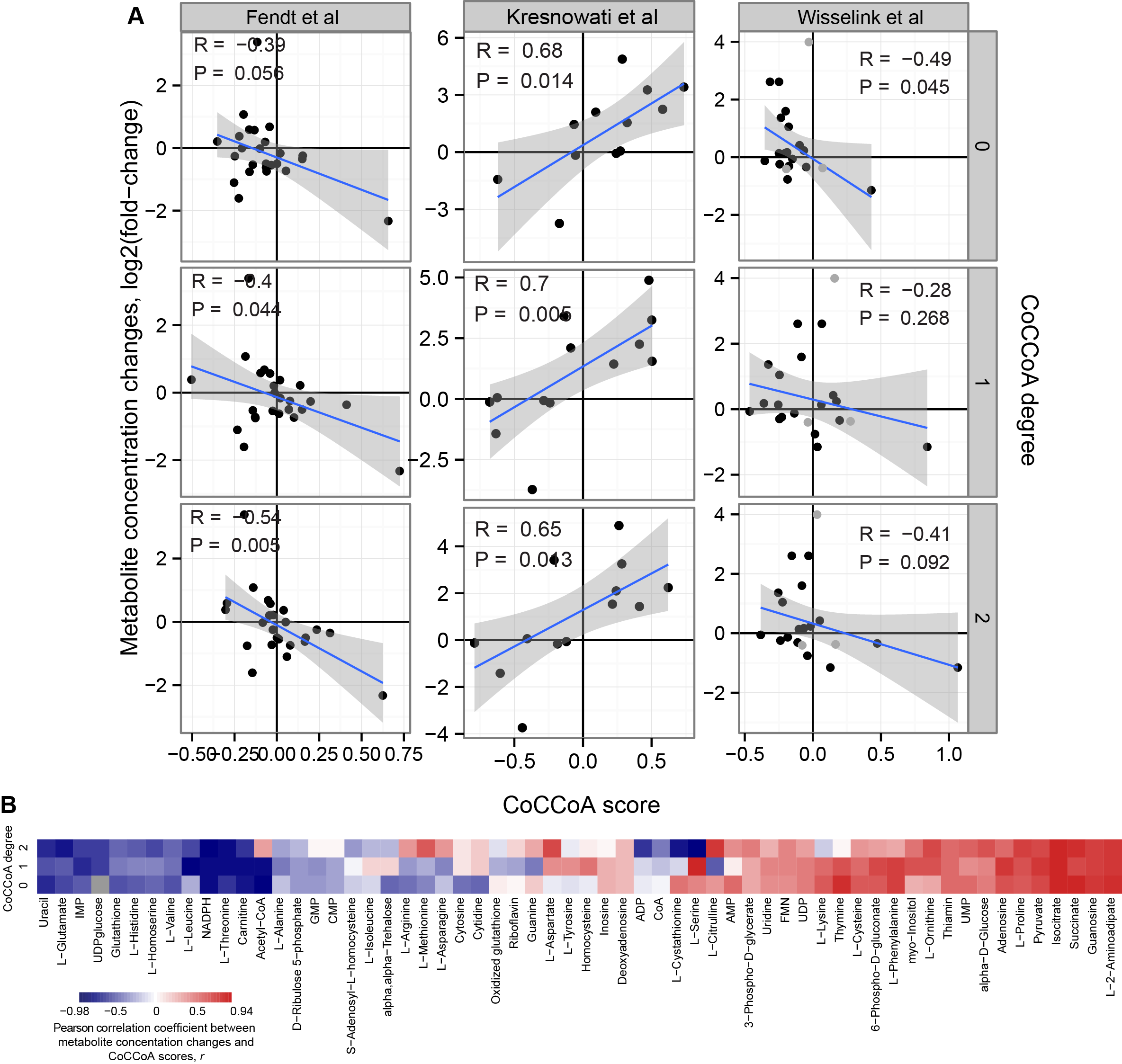

Supplement: Figure S1 — Correlations between experimentally measured metabolite concentration changes and CoCCoA scores based on gene expression fold changes. A) Facet columns correspond to three different pairwise comparison datasets used in our work; rows represent different CoCCoA models. B) Heatmap of Pearson correlation coefficients assessing the applicability of CoCCoA models to the metabolic cycle study. In the present figure, the significance thresholds for the transcript and metabolite fold changes are relaxed in comparison to the data shown in Figure 3B,C in the main text. (PNG) [file pcbi.1003572.s001.png]

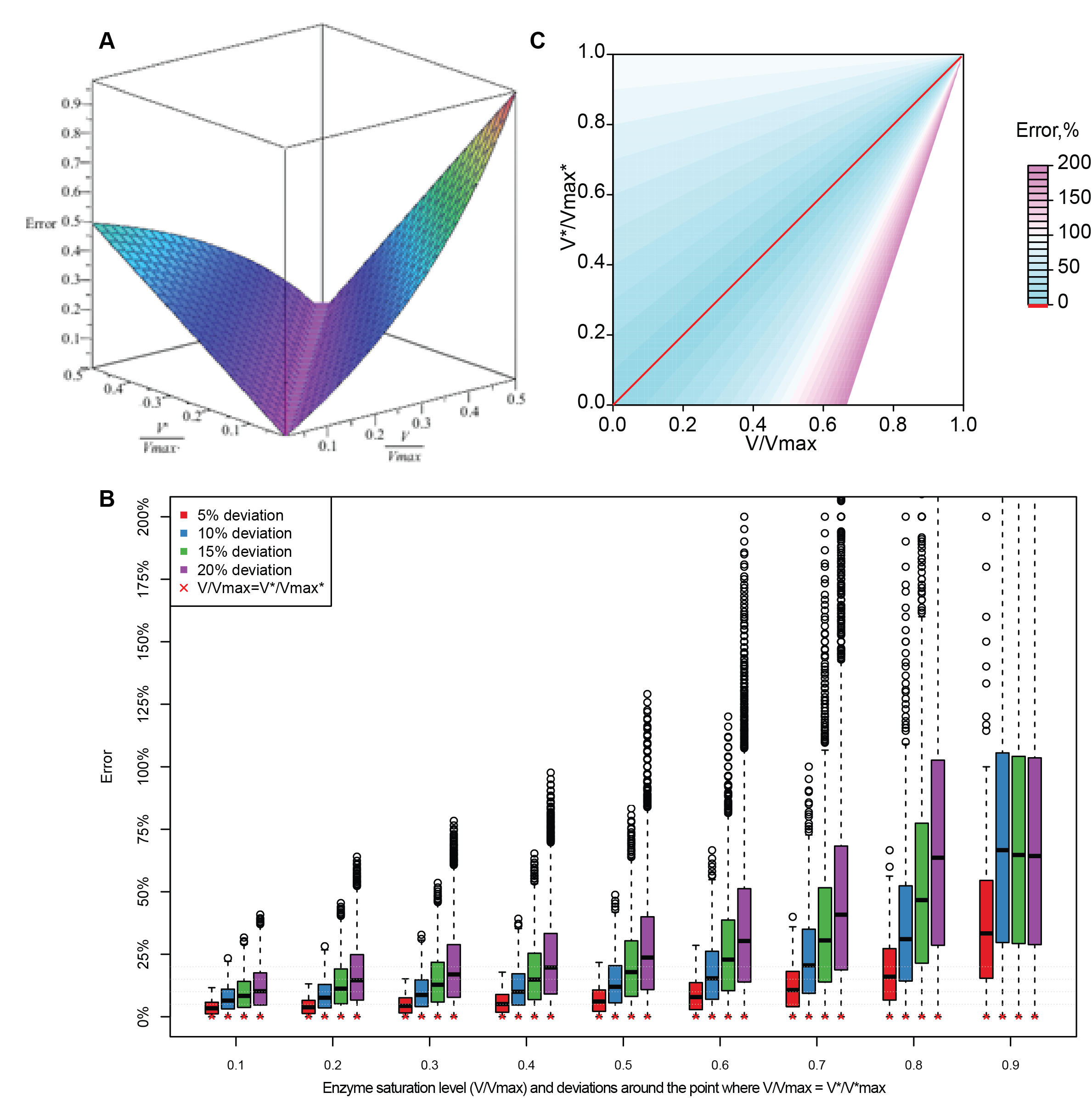

Supplement: Figure S2 — Estimates of error in predicting metabolite concentration changes with MM kinetics when assuming V≪Vmax (see main text for the motivation behind the use of this assumption). A. Error (Z-axis) as a function of V/Vmax and V*/Vmax *. The error function is shown in supplementary Text S1 (equation 11). B. Error estimates around the points where V/Vmax = V*/Vmax *, representing a situation in which the enzyme saturation levels remain unchanged in the perturbed condition. C. 2-D projection of the plot in A, where the errors are represented with different colors. (PNG) [file pcbi.1003572.s002.png]

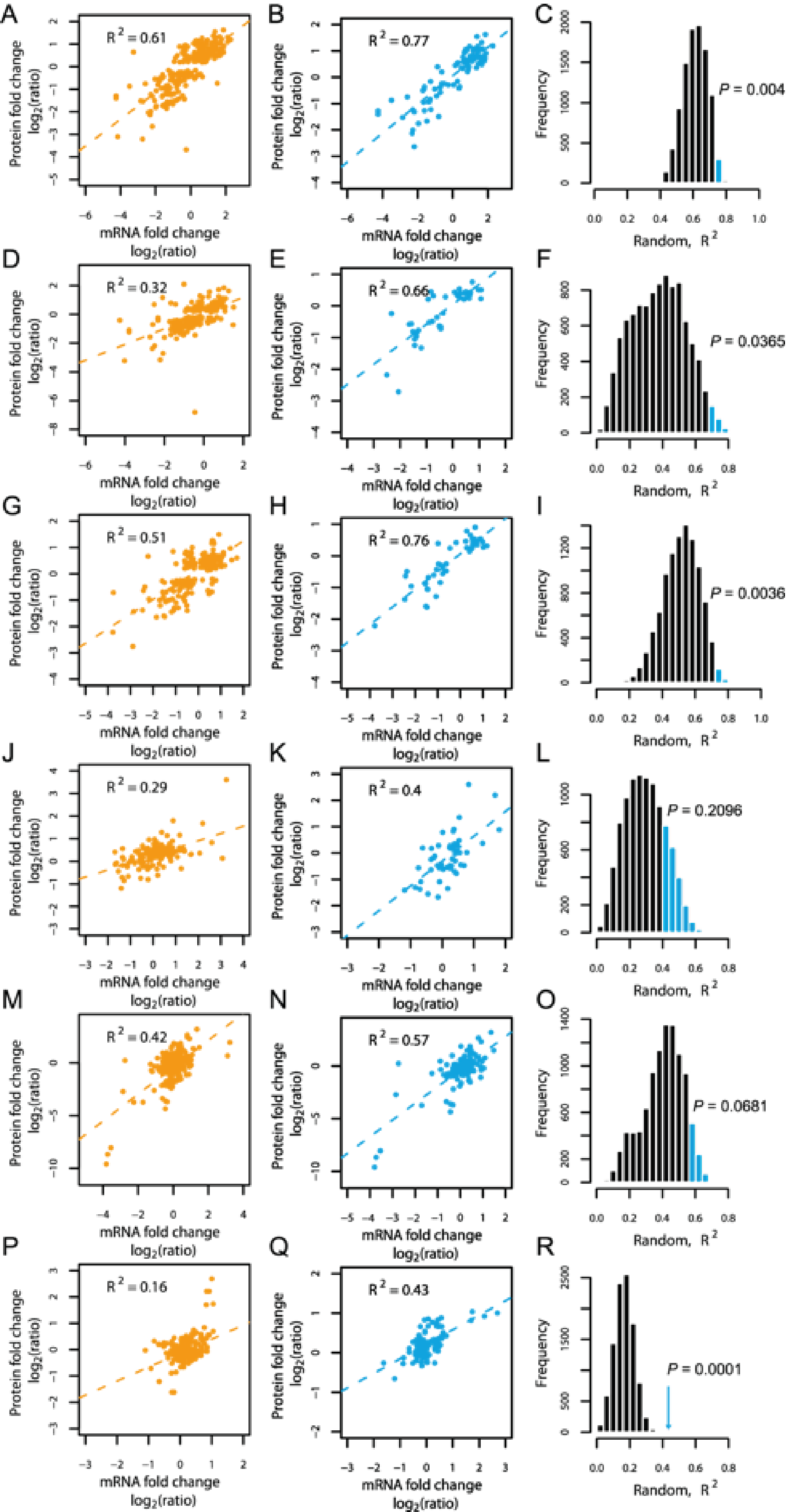

Supplement: Figure S3 — Correlation between protein abundance changes and the corresponding mRNA abundance changes is stronger for metabolic proteins. A, D, G, J, M, P) Correlation including all proteins measured in different datasets. B, E, H, K, N, Q) Correlations including only metabolic proteins (as per genome-scale metabolic model by [17]). C, F, I, L, O, R) Histogram of 10,000 different correlation coefficients obtained for randomly chosen protein-transcript pairs (number of chosen pairs for each correlation being equal to the number of metabolic proteins measured in the corresponding dataset). Blue area denotes the number of random correlations that were higher than those obtained for the correlation based on the actual data. Each row of plots represents a different dataset (from top to bottom), 1, 2, 3 –[38], [51]; 4 –[41]; 5 –[40]; 6 –[41]. (PNG) [file pcbi.1003572.s003.png]

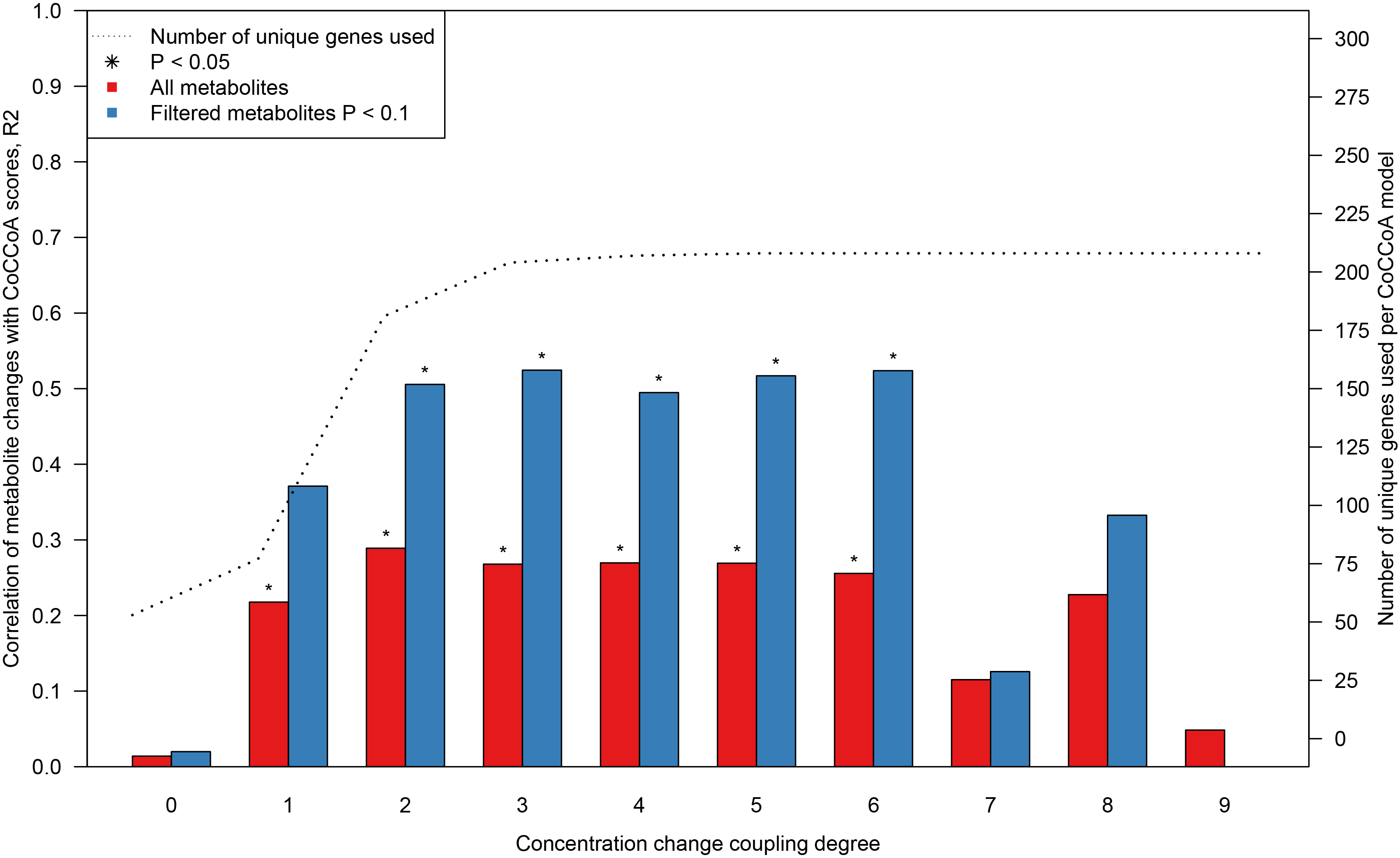

Supplement: Figure S4 — Coefficients of determination for the correlations between experimentally measured metabolite concentration changes and CoCCoA scores corresponding to different degrees. The significance of correlations was assessed against correlations obtained with random permutations of gene labels. (PNG) [file pcbi.1003572.s004.png]

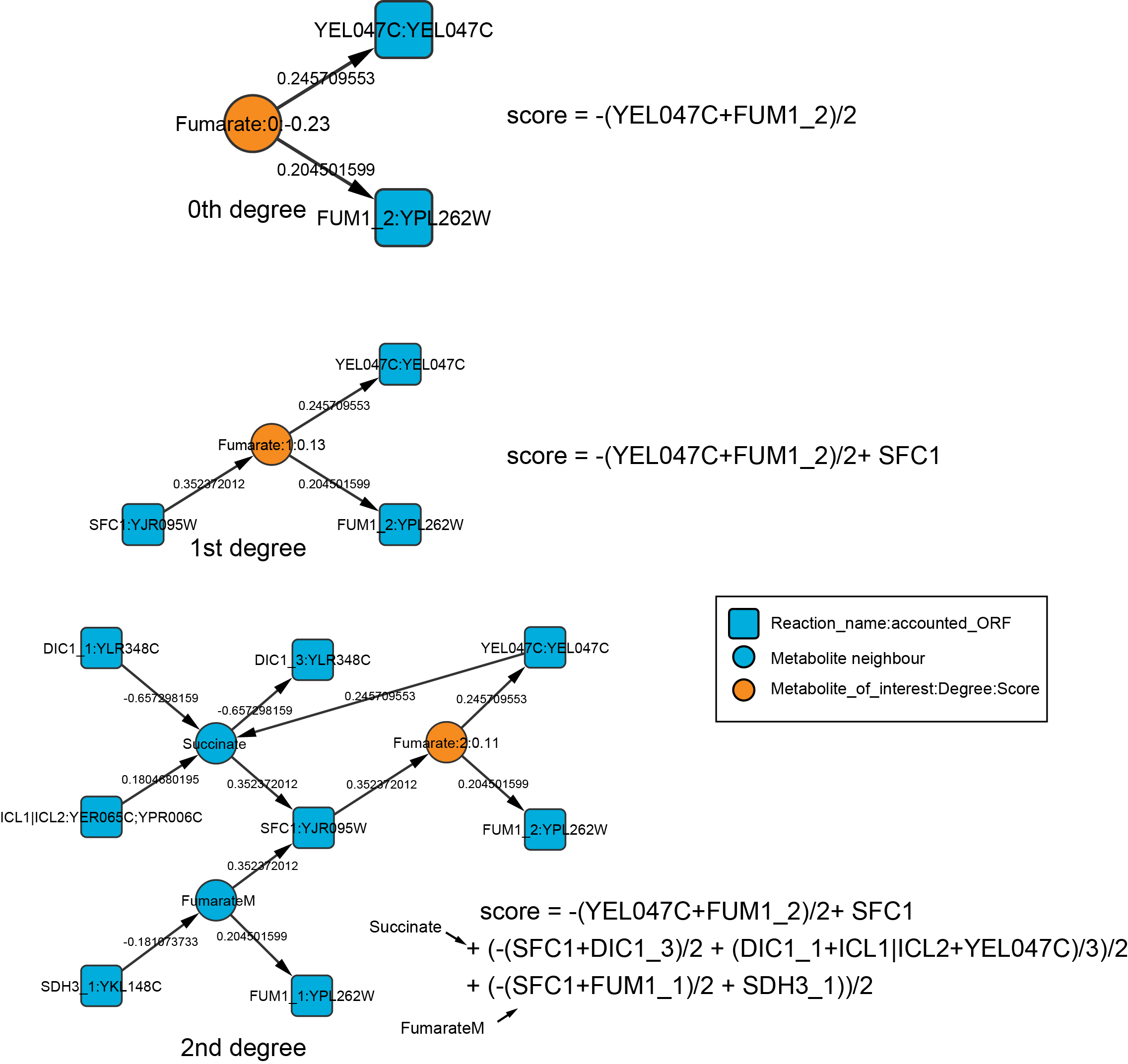

Supplement: Figure S5 — Example CoCCoA score calculations. Shown is the case of fumarate in the Fendt et al. case study. (PNG) [file pcbi.1003572.s005.png]
