# Supplementary material for: Contribution of Network Connectivity in Determining the Relationship between Gene Expression and Metabolite Concentration Changes
Source: PLoS Comput Biol. 2014 Apr 24;10(4):e1003572. doi: 10.1371/journal.pcbi.1003572 (PMC3998873; doi:10.1371/journal.pcbi.1003572)
Supplement: Table S1 — Summary of the growth conditions from the three pairwise comparison case studies used in our analysis. (DOCX) [file pcbi.1003572.s006.docx]

**Table S1** Summary of the growth conditions from the three pairwise comparison case studies and metabolic cycle dataset used in our analysis.

| **Case study #** | **Reference** | **Growth conditions** | **Short description** | **Comparison** | **Remarks** |
| --- | --- | --- | --- | --- | --- |
| 1 | Fendt *et al.* [[7](#_ENREF_7)] | Batch (shake flask), aerobic, glucose minimal medium | Compares Gcr2p null mutant with reference yeast strain. Gcr2p responsible for activation of glycolysis. Measurements are taken at exponential growth phase. | ∆gcr2 mutant *vs* wild-type yeast | Growth rate of mutant was 30% slower than wild-type FY4. |
| 2 | Krewnowati *et al.* [[8](#_ENREF_8)] | Chemostat, aerobic, glucose limited | Growing glucose-limited chemostat culture is subjected to glucose pulse. Data is collected at different time points before and after pulse. | 300s after glucose pulse *vs* time before pulse 0s | Time point of 300s was chosen so as to account for time needed for transcription to affect metabolite levels. |
| 3 | Wisselink et al. [[9](#_ENREF_9)] | Chemostat, anaerobic, arabinose and glucose limited | Evolutionary adapted strain able to grow on arabinose was grown on either arabinose or glucose. | Growth on arabinose of adapted vs growth on glucose of adapted strain | For 0th degree concentration change coupling only 2 points could potentially be used for correlation analysis (no significantly changed transcripts in consumption reactions) |
| 4 | Tu *et al.*[[10](#_ENREF_10),[11](#_ENREF_11)] | Chemostat, aerobic, glucose limited | Saturated batch culture was starved  for ~ 4 h. Continuous culture was then initiated by the constant infusion of  media containing 1% glucose at a dilution rate of ~0.09-0.1 h^-1^ | 11 comparisons against the reference time point marked in Figure 4D | - |
