# Supplementary material for: Contribution of Network Connectivity in Determining the Relationship between Gene Expression and Metabolite Concentration Changes
Source: PLoS Comput Biol. 2014 Apr 24;10(4):e1003572. doi: 10.1371/journal.pcbi.1003572 (PMC3998873; doi:10.1371/journal.pcbi.1003572)
Supplement: Table S2 — Physiological data from the pairwise comparison case study 1. (DOCX) [file pcbi.1003572.s007.docx]

**Table S2** Physiological data from the pairwise comparison case study 1 [[7](#_ENREF_7)]. Comma-separated values denote lower and upper bounds used for constraining the corresponding fluxes. The same constraints were used for metabolic cycle data [[10](#_ENREF_10),[11](#_ENREF_11)].

| **Reaction (mmol/g/h)** | ***∆gcr2*** | **WT** |
| --- | --- | --- |
| Glucose uptake | 10, 11 | 16.15, 17.85 |
| Ethanol secretion rate | 14, 15 | 20, 22 |
| Glycerol secretion rate | 1.66, 2.07 | 2, 2.2 |
| Acetate secretion rate | 0.5,0.7 | 0.75, 1.51 |
| Pyruvate secretion rate | 0.04, 0.06 | 0.06, 0.09 |
| Growth rate | 0.2,0.26 | 0.3, 0.33 |
