# Supplementary material for: Contribution of Network Connectivity in Determining the Relationship between Gene Expression and Metabolite Concentration Changes
Source: PLoS Comput Biol. 2014 Apr 24;10(4):e1003572. doi: 10.1371/journal.pcbi.1003572 (PMC3998873; doi:10.1371/journal.pcbi.1003572)
Supplement: Table S3 — Physiological data from the pairwise comparison case study 2. (DOCX) [file pcbi.1003572.s008.docx]

**Table S3.** Physiological data from the pairwise comparison case study 2 [[8](#_ENREF_8)]. Parameters were estimated for the 300s time point based on the plots provided in the original publication. Comma-separated values denote lower and upper bounds used for constraining the corresponding fluxes constraints.

| **Reaction (mmol/g/h)** | **300s** | **0s** |
| --- | --- | --- |
| Glucose uptake | 3.6, 3.8 | 0.45, 0.6 |
| Oxygen uptake | 4.2, 4.4 | 1.55, 1.8 |
| Ethanol secretion rate | 2.75, 2.9 | - |
| Acetate secretion rate | 0.5, 0.6 | - |
| Glycerol secretion rate | 0.14, 0.16 | - |
| Growth rate | 1.66, 2.07 | 0.04, 0.048 |
