# Supplementary material for: Contribution of Network Connectivity in Determining the Relationship between Gene Expression and Metabolite Concentration Changes
Source: PLoS Comput Biol. 2014 Apr 24;10(4):e1003572. doi: 10.1371/journal.pcbi.1003572 (PMC3998873; doi:10.1371/journal.pcbi.1003572)
Supplement: Table S4 — Physiological data from the pairwise comparison case study 3. (DOCX) [file pcbi.1003572.s009.docx]

**Table S4.** Physiological data from the pairwise comparison case study 3 [[9](#_ENREF_9)]. Comma-separated values denote lower and upper bounds used for constraining the corresponding fluxes constraints.

| **Reaction (mmol[CmolDW]^-1^ h^-1^)** | **Arabinose** | **Glucose** |
| --- | --- | --- |
| “Arabinose”*/Glucose uptake | 61, 62 | 59, 63 |
| Ethanol secretion rate | 91.1, 104.5 | 96, 108 |
| CO_2_ secretion rate | 97, 110 | 100, 112.8 |
| Succinate secretion rate | 0.29, 0.37 | 0.2, 0.5 |
| Glycerol secretion rate | 6.85, 7.15 | 6.9, 7.9 |
| Acetate secretion rate | 0.45, 0.81 | 0.376, 0.42 |
| Pyruvate secretion rate | 0.046, 0.054 | 0.066, 0.074 |
| Growth rate | 1.6, 1.66 | 1.45, 1.55 |

*Growth on glucose was used to estimate reaction directions. Note: CoCCoA cannot be used if many reaction directions change between the two conditions tested. In this case, however, according to Wisselink *et a l*[[9](#_ENREF_9)], the differences in reaction directions are confined to only few fluxes.
