# Supplementary material for: Contribution of Network Connectivity in Determining the Relationship between Gene Expression and Metabolite Concentration Changes
Source: PLoS Comput Biol. 2014 Apr 24;10(4):e1003572. doi: 10.1371/journal.pcbi.1003572 (PMC3998873; doi:10.1371/journal.pcbi.1003572)
Supplement: Table S5 — Reaction directions used for the case studies 1 and 2. (DOCX) [file pcbi.1003572.s010.docx]

**Table S5.** Reaction directions used for the case studies 1 [[7](#_ENREF_7)] and 2 [[8](#_ENREF_8)]. Glycolytic flux directions were fixed according to Fendt *et al*(2).[[7](#_ENREF_7)] For other reactions, directions based on other literature indications were used.

| **Reaction name in the model** | **Reaction** | **Remarks** |
| --- | --- | --- |
| *Glycolysis* |  |  |
| PGI1_1 | alpha-D-Glucose 6-phosphate -> beta-D-Fructose 6-phosphate |  |
| PGI1_2 | alpha-D-Glucose 6-phosphate -> beta-D-Glucose 6-phosphate |  |
| PGI1_3 | beta-D-Glucose 6-phosphate -> beta-D-Fructose 6-phosphate |  |
| FBA1 | beta-D-Fructose 1,6-bisphosphate -> D-Glyceraldehyde 3-phosphate + Glycerone phosphate |  |
| TDH1\|TDH2\|TDH3 | D-Glyceraldehyde 3-phosphate + NAD+ + Orthophosphate -> 3-Phospho-D-glyceroyl phosphate + NADH |  |
| PGK1 | 3-Phospho-D-glyceroyl phosphate + ADP -> 3-Phospho-D-glycerate + ATP |  |
| GPM1_1 | 3-Phospho-D-glyceroyl phosphate -> 2,3-Bisphospho-D-glycerate |  |
| GPM1_2\|GPM2\|GPM3 | 3-Phospho-D-glycerate -> 2-Phospho-D-glycerate |  |
| ENO1\|ENO2\|ERR1_1\|ERR1_2\|ERR2 | 2-Phospho-D-glycerate -> Phosphoenolpyruvate |  |
| ACO1\|YJL200C | CitrateM -> IsocitrateM |  |
| LSC1 | ATPM + CoAM + ItaconateM -> ADPM + Itaconyl-CoAM + OrthophosphateM |  |
| LSC2 | ATPM + CoAM + SuccinateM -> ADPM + OrthophosphateM + Succinyl-CoAM |  |
| FUM1_1 | FumarateM -> MalateM |  |
| FUM1_2 | Fumarate -> Malate |  |
| *Amino acid metabolism* |  |  |
| SHM1 | L-SerineM + TetrahydrofolateM -> 5,10-MethylenetetrahydrofolateM + GlycineM |  |
| SHM2 | L-Serine + Tetrahydrofolate -> 5,10-Methylenetetrahydrofolate + Glycine |  |
| GDH2 | L-Glutamate + NAD+ -> 2-Oxoglutarate + NADH + NH3 | Reaction removed |
| OAC1 | Oxaloacetate -> H+M + OxaloacetateM |  |
| DIC1_1 | Malate + SuccinateM -> MalateM + Succinate |  |
| YDR111C | L-Glutamate + Pyruvate -> 2-Oxoglutarate + L-Alanine |  |
| YFL030W | Glycine + Pyruvate -> Glyoxylate + L-Alanine |  |
| CTP1_1 | Citrate + MalateM -> CitrateM + Malate |  |
