# Supplementary material for: Contribution of Network Connectivity in Determining the Relationship between Gene Expression and Metabolite Concentration Changes
Source: PLoS Comput Biol. 2014 Apr 24;10(4):e1003572. doi: 10.1371/journal.pcbi.1003572 (PMC3998873; doi:10.1371/journal.pcbi.1003572)
Supplement: Text S1 — Supporting text. (DOCX) [file pcbi.1003572.s011.docx]

**Supporting Text S1**

## 0^th^ degree concentration change coupling


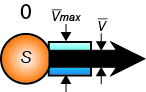


The Michaelis-Menten kinetics equation is given by: $V=\frac{V_{\max}S}{K_{M}+S}$

Rearranging for S gives:

$S=\frac{VK_{M}}{V_{\max}-V}$ (1)

For the reference condition: $S^{*}=\frac{V^{*}K_{M}}{V_{\max}^{*}-V^{*}}$

Dividing Eq.1 by the reference condition equation gives:

$\frac{S}{S^{*}}=\left(\frac{V}{V^{*}}\right)\left(\frac{V_{\max}^{*}-V^{*}}{V_{\max}-V}\right)$ (2)

Assuming that V<<V_max_ & V*<<V_max_* (see main text for the discussion on the validity of the assumption) one gets:

$\frac{S}{S^{*}}=\frac{V}{V^{*}}\frac{V_{\max}^{*}}{V_{\max}}$ (3)

Transforming to log-space Eq. 3 becomes:

$$\ln\frac{S}{S^{*}}=\ln\frac{V}{V^{*}}+\ln\frac{V_{\max}^{*}}{V_{\max}}$$

since V_max_ = k_2_[E], where k_2_ and [E] are, respectively, substrate to product conversion rate and concentration of active enzyme. Assuming that E ∝ T, where T is transcript abundance, and assuming that k_2_ does not change between the two conditions, one gets:

$$\ln\frac{S}{S^{*}}=\ln\frac{V}{V^{*}}+\ln\frac{T^{*}}{T}$$

or

$\ln\frac{S}{S^{*}}=-\ln\frac{T}{T^{*}}+\ln\frac{V}{V^{*}}$ (4)

Eq. 4 is defined as 0^th^ degree concentration change coupling.

## 1^st^ degree concentration change coupling^§^


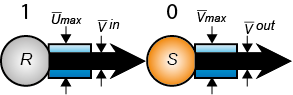


At steady state V_in_ =V_out_. Consequently,

$$\frac{V_{in}}{V_{in}^{*}}=\frac{V_{out}}{V_{out}^{*}}$$

Substituting the flux ratio in the above equation with metabolite and transcript ratios, as given by Eq.4, we obtain:

,

Since the consuming reaction of R is the same as the production reaction of S, $T_{cons}^{R}=T_{prod}$.

Rearranging for S therefore gives:

$\ln\frac{S}{S^{*}}=-\ln\frac{T^{cons}}{T^{cons*}}+\ln\frac{T^{prod}}{T^{prod*}}+\ln\frac{R}{R^{*}}$ (5)

Eq. 5 is defined as 1^st^ degree concentration change coupling.

^§^In the presented scheme, U_max_ is equivalent to $\mathbf{V}_{\mathbf{production}}^{\mathbf{max}}$

## 2^nd^ degree concentration change coupling^§^

##
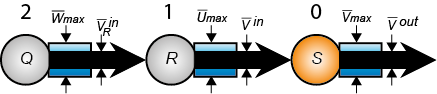


Applying the first degree CoCCoA (Eq.5) to metabolite R, gives:

$\ln\frac{R}{R^{*}}=-\ln\frac{T_{R}^{cons}}{T_{R}^{cons*}}+\ln\frac{T_{R}^{prod}}{T_{R}^{prod*}}+\ln\frac{Q}{Q^{*}}$ (6)

Since$\ln\frac{T_{R}^{cons}}{T_{R}^{cons*}}=\ln\frac{T^{prod}}{T^{prod*}}$, substituting the *R* term from Eq.5 into Eq.6 gives:

$\ln\frac{S}{S^{*}}=-\ln\frac{T^{cons}}{T^{cons*}}+\ln\frac{T_{R}^{prod}}{T_{R}^{prod*}}+\ln\frac{Q}{Q^{*}}$ (7)

Eq. 7 is defined as 2^nd^ degree concentration change coupling.

^§^In the presented scheme, *U_max_* is equivalent to $\boldsymbol{V}_{\boldsymbol{prod}}^{\boldsymbol{max}}$ and *W_max_* is the capacity constraint for the production of metabolite R

## Multiple reactions connected to S

### 0^th^ degree concentration change coupling


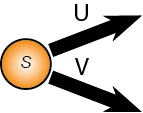


Two reactions using the same substrate *S*. *U* and *V* denote fluxes through the two reactions.

Consider two reactions (carrying flux *U* and *V*) that use the same substrate *S*. For each reaction, 0^th^ degree concentration change coupling (Eq.4) can be applied independently

$$\left\{\begin{matrix} \ln\frac{S}{S^{*}}=-\ln\frac{T_{U}}{T_{U}^{*}}+\ln\frac{U}{U^{*}} \\ \ln\frac{S}{S^{*}}=-\ln\frac{T_{V}}{T_{V}^{*}}+\ln\frac{V}{V^{*}} \end{matrix}\right.$$

The above system of two equations can be summed and rearranged as:

$\ln\frac{S}{S^{*}}=\frac{1}{2}\left(-\ln\frac{T_{U}}{T_{U}^{*}}+\ln\frac{U}{U^{*}}-\ln\frac{T_{V}}{T_{V}^{*}}+\ln\frac{V}{V^{*}}\right)$ (8)

For more than two reactions, similar analysis will imply averaging of fold changes of the corresponding transcripts.

### 1^st^ degree concentration change coupling


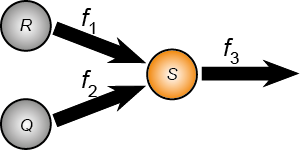


Multiple reactions producing the same metabolite *S*.

At steady state:

$$f_{1}+f_{2}=f_{3}$$

Comparing to the reference condition:

$$\frac{f_{1}+f_{2}}{f_{1}^{*}+f_{2}^{*}}=\frac{f_{3}}{f_{3}^{*}}$$

$$\ln\left(\frac{f_{1}+f_{2}}{f_{1}^{*}+f_{2}^{*}}\right)=\ln\frac{f_{3}}{f_{3}^{*}}$$

Define *f_1_*=*αf_2_,* *β = 1/α*, *f_1_^*^*=*α^*^f_2_^*^* and *β^*^= 1/α^*^*:

$$\ln\left(\frac{f_{1}(1+\alpha)}{f_{1}^{*}(1+\alpha^{*})}\right)=\ln\frac{f_{3}}{f_{3}^{*}}$$

In cases where *α=α** and *β=β**, meaning that the split ratio of fluxes between conditions is unchanging (for example, as suggested in [[1](#_ENREF_1)]), we obtain:

$$\left\{\begin{matrix} \ln\frac{f_{1}}{f_{1}^{*}}=\ln\frac{f_{3}}{f_{3}^{*}} \\ \ln\frac{f_{2}}{f_{2}^{*}}=\ln\frac{f_{3}}{f_{3}^{*}} \end{matrix}\right.$$

$\ln\frac{f_{3}}{f_{3}^{*}}=\frac{1}{2}\left(\ln\frac{f_{2}}{f_{2}^{*}}+\ln\frac{f_{1}}{f_{1}^{*}}\right)$ (9)

Rearrangement of Eq.9 using Eq.4 gives:

$\ln\frac{S}{S^{*}}=\frac{1}{2}\left(\ln\frac{T_{f1}}{T_{f1}^{*}}+\ln\frac{R}{R^{*}}+\ln\frac{T_{f2}}{T_{f2}^{*}}+\ln\frac{Q}{Q^{*}}\right)-\ln\frac{T_{f3}}{T_{f3}^{*}}$ (10)

Equation 10 was used as a basis for calculation of the 1^st^ degree concentration change couplings.

### 1^st^ degree concentration change coupling with protein-mRNA correlation correction factor

$$\ln\frac{S}{S^{*}}=-\beta_{cons}\ln\frac{T^{cons}}{T^{cons*}}+\beta_{prod}\ln\frac{T^{prod}}{T^{prod*}}+\ln\frac{R}{R^{*}}$$

Each transcript change term $\ln\frac{T}{T^{*}}$ was multiplied by a correction factor *β*, which was randomly sampled from a normal distribution with mean and variance estimated based on the values of the slopes of the least squares regression lines for the mRNA-protein fold change data (Supplementary Figure S2).

## Alternative CoCCoA formulation

An alternative formulation of the higher-degree CoCCoA equations includes information from all the intermediate reaction steps till the desired degree. This formulation takes in to account mass balance around metabolites within the desired distance from the metabolite of interest. An example for this formulation is illustrated below where three upstream and one downstream degrees are considered.


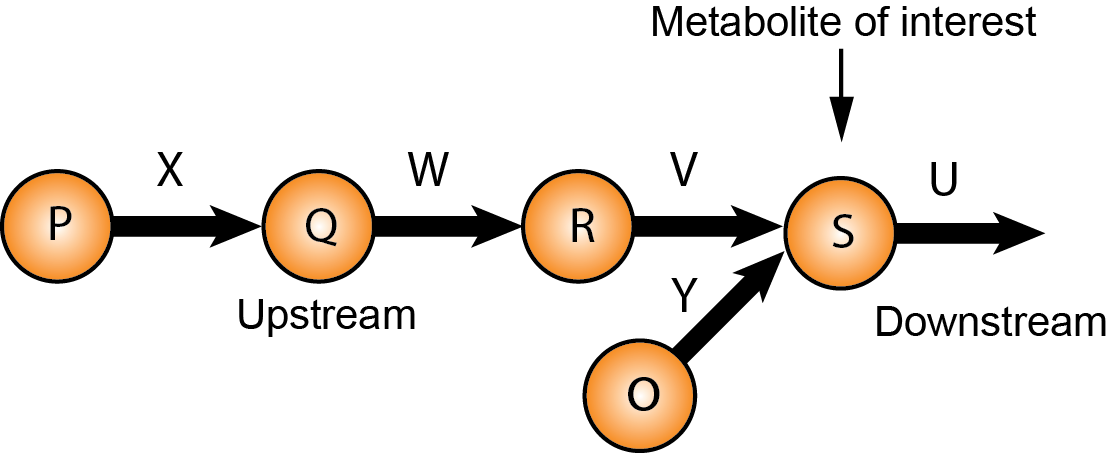

$$\left\{\begin{matrix} \frac{U}{U*}=\frac{V+Y}{V*+Y*} \\ \frac{U}{U*}=\frac{W+Y}{W*+Y*} \\ \frac{U}{U*}=\frac{X+Y}{X*+Y*} \end{matrix}\right.$$

The above system in log space can be rewritten as:

$$\ln\frac{U}{U*}=\frac{1}{3}\ln\left(\frac{V+Y}{V*+Y*}\right)+\frac{1}{3}\ln\left(\frac{W+Y}{W*+Y*}\right)+\frac{1}{3}\ln\left(\frac{X+Y}{X*+Y*}\right)$$

Combining the above equation with Eq. 4 and applying the same assumptions as in Eq. 9 gives:

$\begin{matrix} \ln\frac{S}{S*}=\frac{1}{6}\left(\ln\frac{T_{V}}{T_{V}*}+\ln\frac{T_{W}}{T_{W}*}+\ln\frac{T_{X}}{T_{X}*}+3\ln\frac{T_{Y}}{T_{Y}*}\right) \\ +\frac{1}{6}\left(\ln\frac{R_{V}}{R_{V}*}+\ln\frac{Q_{W}}{Q_{W}*}+\ln\frac{P_{X}}{P_{X}*}+3\ln\frac{P_{X}}{P_{X}*}\right)-\ln\frac{U}{U*} \end{matrix}$ (9)

The above-described method can be applied in the same fashion to derive the equations including the pathways downstream from the metabolite of interest.

### Algorithm for calculation of CoCCoA scores in the alternative formulation

The procedure below describes the algorithm used for computing the scores for the alternative CoCCoA formulation. We note that this algorithm is a heuristic and does not rigorously check for consistency with the mass balances. However, this is of minor concern since several reactions need to be removed from the metabolic network due to the uncertainty in their flux directions, and consequently the final network is not necessarily flux balanced.

INPUT:

CoCCoA_degree ∈ N, where N = {|a| > 0: a ∈ Z}

Bipartite directed graph G = (U,V,E) of metabolic network where U is a set of metabolite nodes, V is a set of reactions nodes and E set of edges between them.

Direction of scoring D = {upstream or downstream}

RFC – dictionary where keys are reactions and values are fold-change of reaction

MFC - dictionary where keys are metabolites and values are fold-change of metabolite (optional)

RFC.keys ∈ V, MFC.keys ∈ U

OUTPUT:

scores for all metabolites at given distance

G^U^ = get_unipartite_graph(G, nodetype=metabolites) //returns a directed unipartite projection (metabolite graph)

IF *direction* is upstream

G^U^ = reverse(G^U^) #reverses edge direction

FOR each *u* in U,

T = empty dictionary with key-value pairs

FOR each *d* in 1:CoCCoA_degree

*targets* = find_target_nodes(in= G^U^, from=*u*, at_distance=*d*), *targets* ∈ U

T = (key = *d*, value = *targets*)

PATHS = empty dictionary with key-value pairs

FOR each key-value pair (*d*, *targets*) in T

a)P = find_all_simple_reaction_paths(in=G, from=*u*, to=*targets*, of_length=*d*+1)),

P ≡ $\left\{ \left\langle v_{i1} \right...\left. v_{id+1} \right\rangle_{1},\left\langle v_{j1} \right...\left. v_{jd+1} \right\rangle_{1},\left\langle v_{k1} \right...\left. v_{kd+1} \right\rangle_{n} \right\}{, v}_{in,jn,kn} \in V, n\in N$ //returns: pathways of reactions at distance d

PATHS = (key = *d*, value = P)

FOR each *d* in 1:CoCCOA_degree:

score = 0, metabolite score at distance *d*

PATHS_f = empty dictionary with key-value pairs

PATHS_f = remove_subpathways(from=PATHS, until_distance=*d*), $\forall P_{i}\in{PATHS\_f}:\{J\in P_{i},K\in P_{i+1}:J \notin K\}, i\leq d$, where *i* is distance and PATHS_f ∈ PATHS //if path at *i* distance is a subset of any path at *i*+1 (including *i* itself),then it is removed; pathways at distance more than *d* are removed; returns: key – distance ≤ *d*, value remained unique pathways at distance

R_counts = empty dictionary with key-value pairs

R_counts = count_reactions_in_paths(PATHS_f)//returns: key - reaction, value - number of times it was present in PATHS_f

R_path_lenghts = empty dictionary with key-value pairs

R_path_lenghts = count_reactions_in_paths(PATHS_f)//returns: key – reaction, value – length of

shortest path where reaction was found in PATHS_f

R = empty array

R = get_reactions(from=PATHS_f, until_distance=d), R ∈ V //returns: unique reactions from PATH_f

FOR each *r* in R, R ∈ V

r_weight = R_counts[*r*]/total(PATHS_f.values())/R_path_lenghts[*r*]

IF *r* is in RFC

score += RFC[*r*]*r_weight

IF MFC is not NULL //adds metabolic component

m_neighbors = empty array

IF *direction* is upstream

m_neighbors = get_output_nodes(in=G, source=*r*), m_neighbors ∈ U

ELSE m_neighbors = get_input_nodes(in=G, source=*r*) , m_neighbors ∈ U

FOR each *m* in m_neighbors, *m* ≠ *u*

IF *m* is in MFC

score += MFC[m]*r_weight/length(m_neighbours)

ELSE score += mean(MFC)*r_weight/length(m_neighbours)

b)//subtracts connected fluxes which are not part of PATHS_f

IF *r* was never found last in elements of PATHS_f

m_neighbors = empty array

IF *direction* is upstream

m_neighbors = get_output_nodes(in=G, source=*r*), m_neighbors ∈ U

ELSE m_neighbors = get_input_nodes(in=G, source=*r*), m_neighbors ∈ U

FOR each *m* in m_neighbours, *m* ≠ *u*, *m* is never a neighbor of elements in R

r_neighbors = empty array

IF *direction* is upstream:

r_neighbors = get_input_nodes(of=*m*, from=G), r_neigbours ∈ V & r_neigbours ∉ R

ELSE r_neighbors = get_output_nodes(of=*m*, from=G), r_neigbours ∈ V & r_neigbours ∉ R

(continued 5 indentations)

IF MFC is not NULL

IF *m* is in MFC

score -= MFC[m]*r_weight/length(m_neighbours)

ELSE

score -= mean(MFC)*r_weight/length(m_neighbours)

FOR each *n* in r_neighbours

score -= RFC[r]*r_weight

save score

The above procedure weights fluxes based on the frequency of their appearance in the paths starting from the metabolite of interest and leading to the target nodes at the desired distance. Additionally, all pathway scores are normalized by their lengths. Some of the key considerations from the algorithm implementation are listed below:

1. The procedure accounts for all paths starting from the metabolite of interest and up to the ‘desired distance’ + 1. The extra distance is tracked so as to account for the triangles in the network, e.g. as shown below:


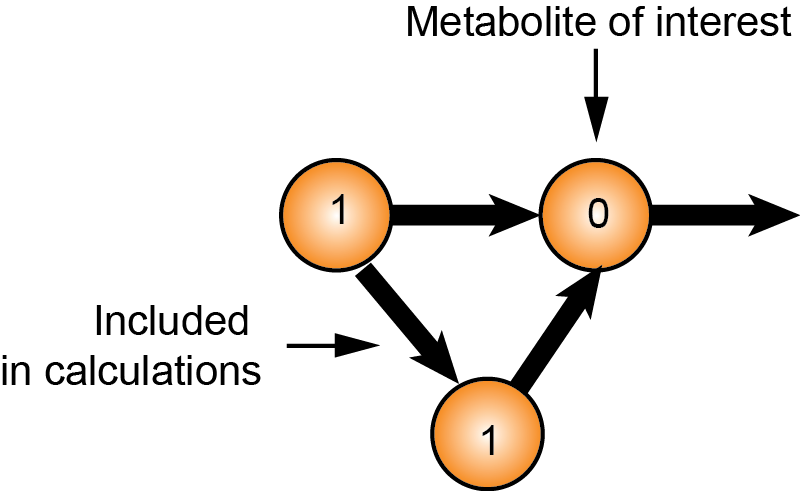


1. The procedure subtracts the contribution of fluxes that are not part of the paths, but are part of the intermediate metabolite mass balances. The ‘last’ metabolites in the pathway are not considered.


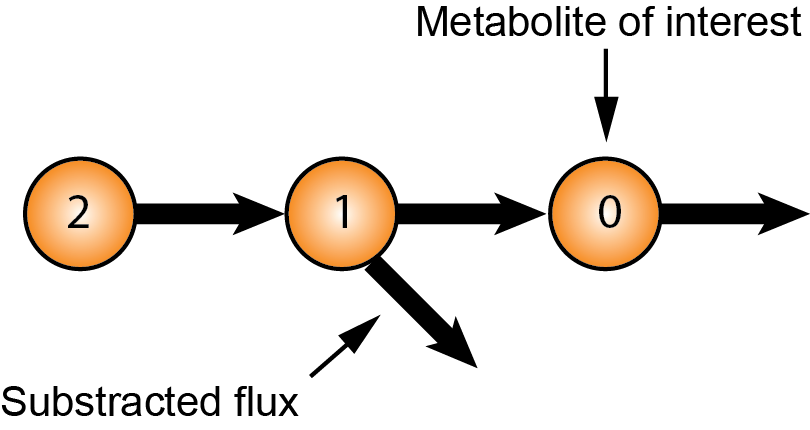


## Error function for Figure S1.

(11)

# References (Supporting information)

1. Haverkorn van Rijsewijk BR, Nanchen A, Nallet S, Kleijn RJ, Sauer U (2011) Large-scale 13C-flux analysis reveals distinct transcriptional control of respiratory and fermentative metabolism in Escherichia coli. Mol Syst Biol 7: 477.

2. Forster J, Famili I, Fu P, Palsson BO, Nielsen J (2003) Genome-scale reconstruction of the Saccharomyces cerevisiae metabolic network. Genome Res 13: 244-253.

3. Usaite R, Nielsen J, Olsson L (2008) Physiological characterization of glucose repression in the strains with SNF1 and SNF4 genes deleted. J Biotechnol 133: 73-81.

4. Usaite R, Wohlschlegel J, Venable JD, Park SK, Nielsen J, et al. (2008) Characterization of global yeast quantitative proteome data generated from the wild-type and glucose repression saccharomyces cerevisiae strains: the comparison of two quantitative methods. J Proteome Res 7: 266-275.

5. Griffin TJ, Gygi SP, Ideker T, Rist B, Eng J, et al. (2002) Complementary profiling of gene expression at the transcriptome and proteome levels in Saccharomyces cerevisiae. Mol Cell Proteomics 1: 323-333.

6. Ideker T, Thorsson V, Ranish JA, Christmas R, Buhler J, et al. (2001) Integrated genomic and proteomic analyses of a systematically perturbed metabolic network. Science 292: 929-934.

7. Fendt SM, Buescher JM, Rudroff F, Picotti P, Zamboni N, et al. (2010) Tradeoff between enzyme and metabolite efficiency maintains metabolic homeostasis upon perturbations in enzyme capacity. Mol Syst Biol 6: 356.

8. Kresnowati MT, van Winden WA, Almering MJ, ten Pierick A, Ras C, et al. (2006) When transcriptome meets metabolome: fast cellular responses of yeast to sudden relief of glucose limitation. Mol Syst Biol 2: 49.

9. Wisselink HW, Cipollina C, Oud B, Crimi B, Heijnen JJ, et al. (2010) Metabolome, transcriptome and metabolic flux analysis of arabinose fermentation by engineered Saccharomyces cerevisiae. Metab Eng 12: 537-551.

10. Tu BP, Kudlicki A, Rowicka M, McKnight SL (2005) Logic of the yeast metabolic cycle: temporal compartmentalization of cellular processes. Science 310: 1152-1158.

11. Tu BP, Mohler RE, Liu JC, Dombek KM, Young ET, et al. (2007) Cyclic changes in metabolic state during the life of a yeast cell. Proc Natl Acad Sci U S A 104: 16886-16891.
